# Differential Regulation of Gonadotropins as Revealed by Transcriptomes of Distinct LH and FSH Cells of Fish Pituitary

**DOI:** 10.3390/ijms22126478

**Published:** 2021-06-17

**Authors:** Lian Hollander-Cohen, Matan Golan, Berta Levavi-Sivan

**Affiliations:** 1 Department of Animal Sciences, The Robert H. Smith Faculty of Agriculture, Food, and Environment, Hebrew University of Jerusalem, Rehovot 76100, Israel; lian.hollander@mail.huji.ac.il; 2 Department of Poultry and Aquaculture, Institute of Animal Sciences, Agricultural Research Organization, Volcani Center, P.O.B 15159, Rishon Letziyon 7505101, Israel; matang@volcani.agri.gov.il

**Keywords:** LH, FSH, pituitary, GPCR, gonadotropins, neuropeptides, cck, GnRH

## Abstract

From mammals to fish, reproduction is driven by luteinizing hormone (LH) and follicle-stimulating hormone (FSH) temporally secreted from the pituitary gland. Teleost fish are an excellent model for addressing the unique regulation and function of each gonadotropin cell since, unlike mammals, they synthesize and secrete LH and FSH from distinct cells. Only very distant vertebrate classes (such as fish and birds) demonstrate the mono-hormonal strategy, suggesting a potential convergent evolution. Cell-specific transcriptome analysis of double-labeled transgenic tilapia expressing GFP and RFP in LH or FSH cells, respectively, yielded genes specifically enriched in each cell type, revealing differences in hormone regulation, receptor expression, cell signaling, and electrical properties. Each cell type expresses a unique GPCR signature that reveals the direct regulation of metabolic and homeostatic hormones. Comparing these novel transcriptomes to that of rat gonadotrophs revealed conserved genes that might specifically contribute to each gonadotropin activity in mammals, suggesting conserved mechanisms controlling the differential regulation of gonadotropins in vertebrates.

## 1. Introduction

The gonadotropin hormones (GtHs), including luteinizing hormone (LH) and follicle stimulating hormone (FSH), are glycoproteins produced by the pituitary gland that are required for normal reproductive function and gonad development in vertebrates. As part of the hypothalamus–pituitary–gonad (HPG) axis, their expression and release are mainly regulated by hypothalamic gonadotropin-releasing hormone (GnRH) and their effects on the gonads are distinct; FSH stimulates the growth of ovarian follicles in females and spermatogenesis in males, while LH induces ovulation in females and spermiogenesis in males [1,2]. The unique secretion pattern of each gonadotropin dictates the reproductive state of the organism and is crucial for maintaining a healthy reproductive state [3].

In mammals, both gonadotropins are secreted from a population of the same cell type, the gonadotroph. The differential secretion of each hormone is regulated by different hypothalamic and systemic hormones. GnRH is considered the main hormone known to directly stimulate differential GtH secretion. It is secreted in a pulsatile manner, where low-frequency GnRH pulses tend to release FSH and high-frequency GnRH pulses stimulate LH release [3,4]. Additional hormones that interact with GnRH signaling, like activin/inhibin, different feedback mechanisms involving gonadal steroids [5], and different autocrine/paracrine/juxtacrine pathways [6] were also shown to regulate the differential secretion of LH and FSH. In teleosts and mammals, it has been recently established that GnRH is part of an orchestra of different neuropeptides that together control the synthesis and release of FSH and LH [7,8]. Different hypothalamic neurohormones were shown to affect gonadotropin secretion like kisspeptin [9,10,11,12,13], neurokinin B (NKB) [14], LPXRFa peptide [9,15], spexin [16], and more, some of which suggest a direct regulation on the gonadotropic cells. Nevertheless, the exact cellular mechanism and hormone regulation that leads to the differential secretion and transcription of LH and FSH remain unknown.

Teleost fish are a particularly attractive model to dissect the differential regulation of GtH synthesis/secretion since, in contrast to mammals, LH and FSH are produced in discrete cell populations. We previously demonstrated the unique spatial organization in the fish pituitary gland of each cell type using transgenic tilapia expressing GFP in FSH cells and mCherry in LH cells [17,18]. LH cells exhibit close cell–cell contacts and form a continuous network throughout the gland, while FSH cells were more loosely distributed but maintained some degree of cell–cell contact by virtue of cytoplasmic processes. The unique spatial distribution of these cell types had also manifested functional differences like gap–junction potentiation of LH secretion across the population of cells in response to GnRH [17].

We took advantage of these discrete LH and FSH populations in teleosts to reveal the transcriptional basis underlying their differential secretion. Using transgenic tilapia with fluorescent-labeled gonadotrophs, we performed RNA-seq on purified LH and FSH cells from the pituitaries of transgenic males and females. We found distinct genes which are enriched in each cell type and reveal new candidates that may directly regulate LH or FSH and different cellular pathways that are unique to each GtH cell type. We also identified conserved genes between mammals and teleosts that specifically regulate LH or FSH cell activity by comparing the fish cell specific gene expression to that of the rat gonadotroph, thereby identifying genes that may suggest functions which are unique to each hormone, allowing them to be synthesized/secreted in a differential manner while being regulated by the same GnRH hormone and secreted from the same cell.

## 2. Results and Discussion

### 2.1. The Emergence of the Mono-Hormonal System from Bi-Hormonal Gonadotrophs

**Figure 1:**
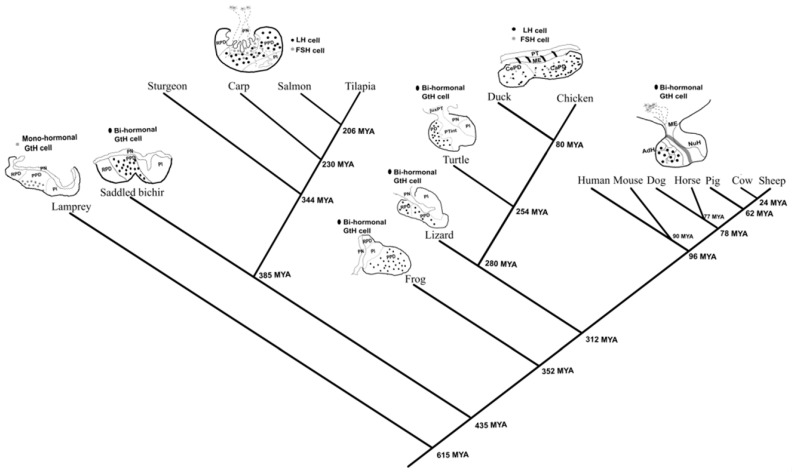
Taxonomic tree of representative vertebrate taxa showing differences in morphology and gonadotroph organization. The distribution of FSH and/or LH cells in the vertebrate pituitary is either bi-hormonal (one cell expressing both gonadotropins), or mono-hormonal cells (two cells secreting each a different gonadotropic hormone). An exception is the proto-glycotrope expressing only one gonadotropic hormone that exists in lamprey. From the different taxa, only the teleost and avian exhibit the mono-hormonal cell morphology while mammals, reptiles, and amphibians exhibit the bi-hormonal morphology. The taxonomic tree was prepared using TIMETREE [25] and visualized with the pituitary diagrams using Inkscape. The evolutionary tree includes only organisms with known information regarding their GtH distribution in the pituitary. Information regarding each organism was obtained from: Mouse (*Mus musculus*) [26,27], dogs (*Canis lupus familiaris*) [28], pigs (*Sus scrofa domesticus*) [29], human (*Homo sapiens*) [30], sheep (*Ovis aries*) [31], horse (*Equus ferus caballus*) [32], cow (*Bos Taurus*) [33], lamprey (*Petromyzon marinus*) [34], Saddled bichir (*Polypterus endlicherii*) [35], tilapia (*Oreochromis niloticus*) [17], carp (*Cyprinus carpio*) [36], salmon (*Salmo salar*) [37], sturgeon (*Acipenser gueldenstaedtii*) [38], turtle (*Geoclemys reevesii*) [39], chicken (*Gallus gallus*) [24], duck (*Anas acuta*) [40], lizard (*Calotes versicolor*) [41], and frog (*Rana japonica*) [42]. Rostral pars distalis (RPD), proximal pars distalis (PPD), pars intermedia (PI), pars nervosa (PN), pars distalis (PD), juxtaneural pars tuberalis (juxPT), pars tuberalis interna (PTint), pars tuberalis (PT), median eminence (ME), cephalic pars distalis (CePD), caudal pars distalis (CaPD), adenohypophysis (AdH), neurohypophysis (NuH).

The pituitary gland is a vertebrate novelty and is derived from the adenohypophyseal neurogenic placode [19]. Among different taxa and classes there are significant variations in the organization of the gonadotropic cells in the pituitary gland (Figure 1). In lampreys, an ancient jawless fish, the pituitary contains a proto-glycotrope secreting two hormones: thyrostimulin and only one glycoprotein hormone [20,21,22]. Although cartilaginous fishes possess two gonadotropins [23], their cellular distribution is unknown. Two different morphological phenotypes of gonadotrophs emerged in later evolved vertebrates, with some lineages evolving bi-hormonal cells (one cell secreting both LH and FSH: amphibians, reptiles, and mammals) and some evolving mono-hormonal cells (two cells secreting each a different gonadotropic hormone: teleost and avian). It is very tempting to think that this phenomenon will have an evolutionary common origin, but it seems that this is a case of convergent evolution since only two very distant vertebrate classes demonstrate the mono-hormonal strategy: teleosts [1] and birds [24] (Figure 1).

Interestingly, both mono-hormonal classes have evolved from bi-hormonal classes. In teleosts, it seems that the mono-hormonal system had evolved somewhere in the course of the actinopterygian radiation, as a fish from the polypteridae (*Sadddled bichir*), a basal family in this class, contain bi-hormonal morphology while a fish from the more advanced acipenseridae family in this class (Sturgeon) exhibit the mono-hormonal morphology, suggesting that the mono-hormonal system might be present in fish that do not belong to the teleost order. Unfortunately, no evidence regarding the gonadotrophs morphology exists in other fish orders except the ones mentioned in Figure 1.

How this unique change in the cell morphology contributes to the reproductive strategy and functionality of these classes via the HPG axis is an enigma. We can speculate that the evolutionary pressure for the segregation of those two cells is either due to a unique functionality of LH or FSH in those organisms or a unique regulatory process of their activity, directly linking between reproduction and different metabolic processes like growth, feeding and homeostasis. Since LH activity in vertebrates is well characterized and essentially conserved [43,44], we suspect that FSH might exhibit an unknown regulatory activity which is unique to teleosts. By revealing conserved genes between a mono-hormonal and a bi-hormonal organism, we were able to contemplate which of the functions identified in each cell type had a common source and which had uniquely evolved within the mono-hormonal system, thereby establishing the basis for understanding the unique functionality and regulation of each hormone in the different lineages.

### 2.2. The Isolation of LH and FSH Cells

**Figure 2:**
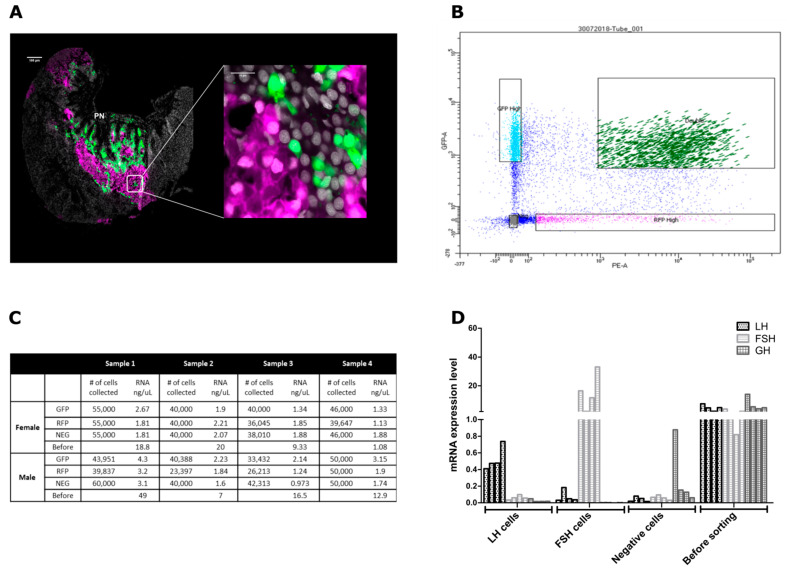
LH and FSH cells isolated from transgenic tilapia expressing double labeled pituitaries. (**A**) Confocal imaging of a double-labeled pituitary slice from mature transgenic Nile tilapia expressing RFP (magenta) under the regulation of LH promotor and GFP (green) under the regulation of FSH promoter. The right image contains a 60x magnification of the marked square in the left image revealing each hormone is expressed in a different cell. DAPI is used for visualizing the cell’s nucleus (gray). (PN) Pars nervosa. Scale bar: 100μM and 10 μM. (**B**) Scatter plot showing separation between GFP and RFP marked cells of the FACS assayed cells that were collected from the double labeled pituitaries. In each FACS assay four fractions were collected: GFP high- FSH positive cells, RFP high- LH positive cells, double—RFP and GFP positive cells, and negative—all non-LH or FSH pituitary cells. For each FACS assay, between 16 and 21 pituitaries were collected and each assay was repeated four times for each sex. The gonadosomatic index of fish used for each assay is detailed in Supplementary Figure S1. The complete gating parameters of the FACS assays are available in Supplementary Figure S2. (**C**) The number of collected cells and the amount of RNA extracted from each fraction of the FACS cells. (**D**) Real-time PCR validation of lhb, fshb, and gh in the “before” and “after” FACS fractions revealing LH and FSH enrichment in each fraction respectively, each line representing a FACS fraction (*n* = 1). Values were normalized against the reference gene EF1α using the comparative threshold cycle method (−∆CT). The primer sequences used for real-time PCR are available in Supplementary Table S1.

Confocal imaging of double-labeled pituitary expressing RFP in LH cells and GFP in FSH cells confirmed the expression of the fluorophores in the proximal pars distalis (PPD), (Figure 2A) where each fluorophore was expressed exclusively in different cell types. FSH cells are located dorsally to the LH cells closer to the dorsal projections of the pars nervosa (PN) and both cell types are distributed from the ventral to the posterior side of the pituitary as was shown before for mono-labeled transgenic fish [17,45]. Even though fish pituitary morphology is very distinct, its development, major compartments, and functions within the HPG axis are well conserved with other vertebrates [46,47].

In order to achieve specific separation of LH or FSH expressing cells, we used FACS to sort the double labeled pituitaries. Four fractions of cell populations were collected (Figure 2B): GFP-positive FSH cells only, RFP-positive LH cells only, negative (non-fluorescent) cells, and RFP and GFP positive double-labeled cells. The double-labeled fraction was not subjected to any further analysis since it probably contained doublets of LH and FSH cells. The sorting was performed four times for male pituitaries and four times for female pituitaries, where each fraction contained between 23,397 to 60,000 cells and extracted between 0.97 ng/μL to 4.3 ng/μL total RNA (emerging from 16–20 pituitaries/batch/sex) (Figure 2C). Before each FACS procedure, a sample of 100 μL from the cell solution was collected, which yielded between 1.08 ng/μL to 49 ng/μg total RNA (Figure 2C). Real-time PCR validation of LHβ subunit (*lhβ*), FSHβ subunit (*fshβ*), and growth hormone (*gh*) revealed that the cells were successfully separated (Figure 2D), as their corresponding hormones were enriched in the respective fractions. GH, a highly expressed pituitary hormone [27,48], was expressed only in the negative fraction and in the fraction that contained cells before sorting where all pituitary hormones are expressed. We can therefore conclude that our sorting strategy generated highly enriched LH and FSH cell fractions that allowed us to confidently attribute the molecular signature revealed by the transcriptome analysis to a specific cell population.

### 2.3. Identifying Enriched Genes in Both FSH and LH Cells

**Figure 3:**
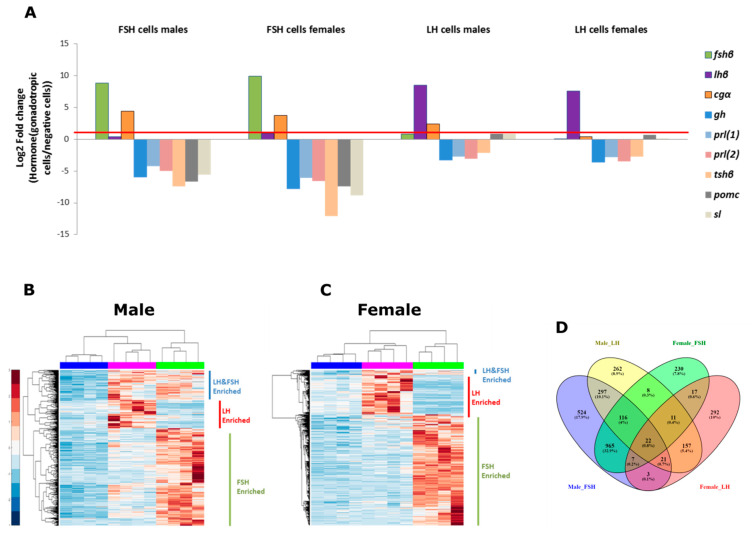
Identifying differentially expressed genes in LH and FSH cells. (**A**) The fold change (represented in log2) of normalized reads of the common pituitary hormone genes in the sorted fractions, as expressed in the RNA-seq libraries. Values are compared to the negative cells fraction. Positive values represent genes enriched in LH and FSH cells and negative values represent genes enriched in the negative cell fraction. Supplementary Table S2 details the exact fold change values for each hormone in each fraction and the gene id for each hormone. As predicted, each hormone (the beta subunits lhβ/fshβ together with the common alpha subunit, cgα, which constructs the complete mature hormones) is highly enriched in its own cell type and no other hormone contains a significant expression value in the gonadotrophs. In order to ensure that only LH or FSH cells specific genes are analyzed in each relevant fraction, we filtered for genes with a fold change greater than 2 (log_2_(1), represented by the red line) for the cluster analysis. (**B**,**C**) Clustergram analysis of normalized reads (FDR corrected *p* < 0.05, log_2_(Fold change) > 1) in LH, FSH, and negative fractions in each repeat (*n* = 4) in males (**B**) and females (**C**). Each repeat from the same fraction was clustered together (top dendrogram). Three main clusters of expressed genes were observed in each clustergram (left dendrogram); genes that were enriched in LH fraction, genes that were enriched in FSH fraction and genes that were enriched in LH and FSH fractions. All the genes used for the clustergram analysis and their values are publicly available in GEO (ID:GSE159470). (**D**) Venn diagram combining the male and female clustergram results, representing the number of enriched genes in each cluster and in each sex revealing high number of genes that are either cell specific or sex specific and only 22 genes that are common to all. The diagram was created using Venny_2.1 (https://bioinfogp.cnb.csic.es/tools/venny/) (accessed on 24 September 2019).

To identify the enriched genes (EG) in each cell type, we compared the expression of genes in each gonadotroph library to the negative library. Due to the high number of genes expressed in LH and FSH cells, we only investigated the genes that are enriched in each gonadotroph type compared to the rest of the cells in the pituitary; therefore genes that are common to LH or FSH and might also be expressed in other cell types are not discussed in the current work. The fold change expression of nine known genes of pituitary hormones were validated (Figure 3A); lhβ, fshβ, glycoprotein α-subunit (*gcα*), gh, two isoforms of prolactin (*prl(1)* and *prl(2)*), thyroid-stimulating hormone (*tshβ*), somatolactin (*sl*), and pro-opiomelanocortin (*pomc*). LH and FSH cells fractions were enriched specifically with their cognate hormones (*lhβ* and *fshβ*, respectively) and the rest of the hormones were highly expressed in the negative fraction.

In addition, three genes had higher expression in the gonadotrophs fractions compared to the negative fraction: *cgα*, *pomc*, and *sl*. As expected, the cgα of the heterodimeric hormones LH and FSH was also enriched in the LH and FSH fraction—but to a lower extent, probably due to the fact that it is also a part of the heterodimeric glycoprotein hormone TSH which is highly expressed in the negative fraction. Surprisingly, *pomc* and *sl* have low fold change expression in the LH fraction; both hormones are complex and less studied in the pituitary glands of teleosts. *Pomc* encodes multiple neuropeptides and has three different subtypes in teleost [49,50]. The *sl* gene is identified only in fish and some amphibians [51], and exhibits different functions in different fish species [52]. Their low expression can be either due to small contamination of these cells in this fraction or an endogenous expression of the hormone in these cells. In order to reduce the chance of cross-contamination, only genes that had a fold change higher than 2 were used in the clustergram analysis to identify EGs in the gonadotroph, thereby ensuring the elimination of genes from unwanted cells contamination in the FACS procedure.

Clustergram analysis of the EGs (Fold change > 2, false discovery rate (FDR) corrected *p* < 0.05) showed a clear separation between the different cell types in male and female (Figure 3B,C, respectively) where the experimental repeats of each cell type were clustered together. We categorized the genes in each sex into three groups: enriched in LH cells, enriched in FSH cells, and enriched in both cell types. In total, males exhibit 1499, 438, and 456 genes uniquely enriched in FSH, LH, and both cell types respectively, and females exhibit 1319, 473, and 57 genes uniquely enriched in FSH, LH, and both cell types, respectively. In both sexes, the FSH group has higher number of enriched genes suggesting that FSH cells differ more from the rest of the pituitary cells.

In a Venn diagram combining the distribution of EGs in the different sexes and cell types (Figure 3D), the FSH fraction had more shared genes between the different sexes (965 shared genes vs. 524 and 230 genes uniquely expressed in males and females, respectively) compared to the LH fraction, which exhibits more unique genes in each sex (292 genes in females and 262 genes in males while only 157 genes are common to both), suggesting that LH cells are more sex-specific than FSH cells. Moreover, the sex- and cell-specific enriched genes can give us a clue regarding the factors that elicit LH-specific or FSH-specific regulation.

### 2.4. Functional Annotation of Enriched Genes in LH and FSH

**Figure 4:**
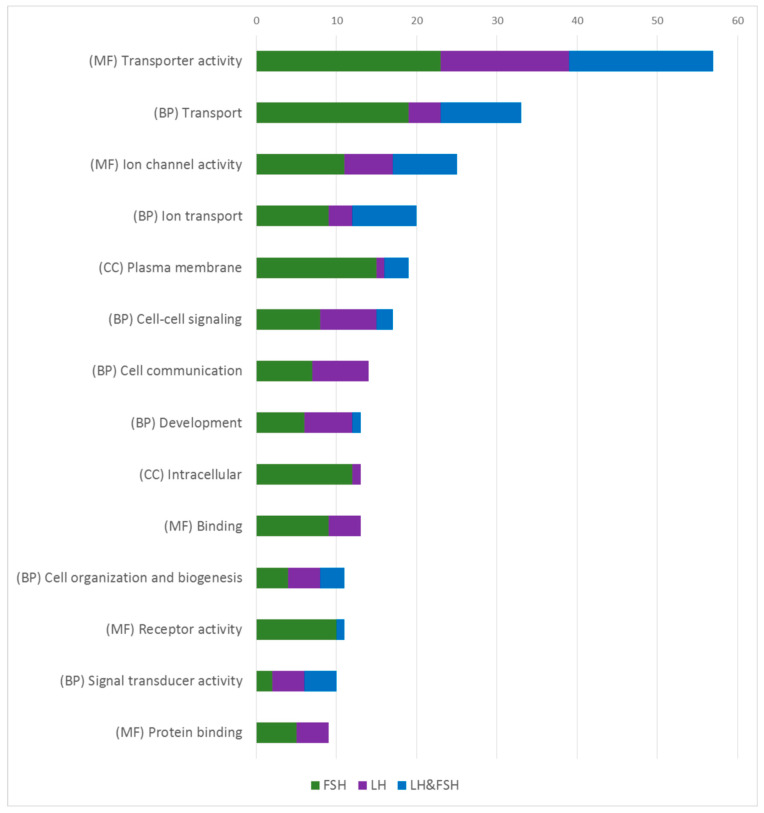
Functional enrichment analysis of enriched genes in each cell type and sex. Categorized GO annotation of functional enrichment analysis of the EG in males and females that were obtained from the clustergram analysis (Figure 2B,C). Values represent the number of GO annotations in each GO group, as each GO group represents the sum of GO annotations in males and female for each fraction (FSH in green, LH in purple, and FSH and LH in blue). The most substantial GO groups are common to both cell types while some express the same genes (blue) and some express genes which are unique to each cell type (FSH in green and LH in purple) revealing how similar LH and FSH cells are in their ontology, and how each cell type acquires its own unique processes to establish their unique functionality. Functional enrichment was performed with GO annotations of molecular functions (MF), biological processes (BP), and cellular component (CC). The exact procedure is detailed in the methods (Section 3.7).

Functional analysis performed on the specifically enriched genes of LH or FSH cells (Figure 4) revealed that the gene ontology (GO) annotations containing the highest amount of functions are common to both LH and FSH cells, supporting the hypothesis that the cells shared a common ancestor, while the different gene repertoires in each cell reveals different mechanisms that allowed for the differential transcription/secretion of each GtH.

The most enriched GO clusters in both cell types are related to channels and ion regulation (like “transporter activity”, “ion channel activity”, “cell–cell signaling” and “plasma membrane”). The excitability of gonadotropic cells and other endocrine cells in the pituitary have been extensively studied over the years [16,53,54,55,56,57,58]. The electrical activity of the gonadotrophs is considered the key factor for the episodic and synchronized activity of those cells [59]. Since most of the studies have been conducted in mammals (where both gonadotropins are secreted from a common cell), the exact electrical properties that differentially regulate LH or FSH secretion and expression remain elusive. In fish, even less is known regarding the different electrical properties of LH and FSH. Different electrical properties between LH and FSH cells have been shown in Atlantic cod (*Gadus morhua*) dispersed pituitary cells, where each cell exhibit different voltage depended current and tail current (KCa) profiles [58]. Additionally, LH and FSH cells exhibit different calcium responses to GnRH stimulations in an intact pituitary and brain preparation of medaka [60]. In our transcriptome analysis, different types of voltage gated channels and calcium channels are enriched in each cell type suggesting different excitability properties regulating LH and FSH.

Additional GO annotation of the enriched genes relates to internal signaling pathways and hormone packaging, (like “binding” and “protein binding”) that include genes that relate to the activity of intracellular proteins like SNARE and GTPase. Interestingly in those GO annotations, each cell is enriched with a unique set of genes. GO annotations that relate to internal signaling pathways (“signal transducer activity”) of hormones and steroid stimuli and GO annotations that include vesicle formation and transport activity (“intracellular”), most of them are highly enriched mainly in FSH cells revealing how those mechanisms are diverse in FSH cells.

### 2.5. Highly Enriched Genes in LH and FSH Cells

By looking at the 10 most enriched genes (EG) in each cell type (Table 1), unique genes that are expressed in each gonadotroph highlighted different functions that might be unique to each. The two most enriched genes are, not surprisingly, the associated gonadotropic hormone of each cell: *lhβ* in LH cells (Males _log2(fold change)_ = 7.6, Females _log2(fold change)_ = 8.5) and *fshβ* in FSH cells (Males _log2(fold change)_ = 8.84, Females _log2(fold change)_ = 9.91).

Additional EG that related to gonadotropin secretion are two types of major sex steroid receptors: estrogen receptor 2 (*esr2*) was enriched in both LH cells (log2(fold change)_male/females_= 5.86/4.35) and FSH (log2(fold change)_males/females_ = 5.41/5.71) while the progesterone receptor (*pgr*) was enriched only in LH cells, mainly in females (log2(fold change)_females_ = 3.63). Steroid hormones produced by the gonads, 17α-20β-dihydroprogesterone (DHP) and estradiol (E2), play an important role in the regulation of reproduction by relaying gonadal growth up the HPG axis in a classic feedback loop [1,61,62]. The unique expression of their receptors in LH and FSH cells correlates with the accepted role of LH and FSH during the reproductive cycle. Both FSH and LH can increase estradiol secretions, which enhance vitellogenin synthesis, however LH primarily increases DHP secretion that enhances final oocyte maturation and ovulation [1,61,63]. Accordingly, esr2 has higher expression in both gonadotropic cells compared to *pgr*, which might indicate a more fundamental regulation of LH and FSH cells activity by estradiol, and a unique regulation of LH cells by DHP.

Metabolic balance is linked directly to reproductive status in vertebrates, where initiation of reproduction is affected by the amount of body energy reserves and its responsiveness to diverse metabolic factors [64,65,66]. Though different neuroendocrine mechanisms responsible for the association between energy balance and reproduction were identified in the hypothalamic center of mammals and fish [67,68,69], our data suggest there are metabolic factors that directly affect gonadotroph activity, especially on FSH cells. One of the top 10 EG that was related to metabolic regulation is the cholecystokinin receptor (*cckr*). CCK is a peptide hormone expressed in the central nervous system and the gut that controls different processes like digestion, satiety, and anxiety [70]. *cckr* had enormous expression in FSH cells (normalized read counts male/female = 20112.98/8826.10) suggesting a direct link between the gastrointestinal hormone CCK and FSH activity. In fish, CCK seems to act directly on the pituitary. An immunohistochemical study showed that CCK neurons from the brain innervate into the proximal pars distalis of the pituitary, and that CCK stimulates LH release in vitro in goldfish [65]. However, in mammals there is no evidence for its direct effect on gonadotroph activity.

Additional metabolic hormones enriched mainly in LH cells, but also in FSH cells, is apelin (*apln*), a novel bioactive peptide that plays pivotal roles in various physiological functions like energy metabolism, food intake, angiogenesis, and neuroendocrine functions [71,72]. In mammals, it is known to suppress LH secretion [73,74] while in fish, *apln* was mainly characterized with regard to feeding regulation [75,76,77] and development [78]. Due to its relatively high fold change expression in LH cells (log2(fold change) = 5.05), it might also exhibit paracrine and endocrine regulation on the HPG axis in fish, mainly on LH activity.

Additional genes that relate to different functions like signal transduction, cell morphology, protein trafficking, transcription factors, and more (Table 1) had been identified in the top 10 EGs. While the exact roll of most of these genes in the gonadotrophs is unknown, the unique expression of known regulatory genes like *esr2*, *pgr*, *cckr,* and *apln* in each cell type reveal differences in the steroid feedback regulation, metabolic regulation, and cell signaling in each cell type.

### 2.6. The Unique GPCR Signatures of Each Gonadotropic Cell

The release of LH and FSH is regulated through neuropeptides that are secreted from the brain and relay their activity through specific receptors that frequently belong to the GPCR family [1]. Identification of different endocrine regulators of LH or FSH secretion has been a challenging task often attempted by identifying specific changes in a mixed cell population or in a bi-hormonal population of cells. Though GnRH is the main neuropeptide regulating both GtHs’ secretion in vertebrates [1], identifying additional GPCRs of novel peptides that are differentially expressed in each GtH cell can reveal the unique regulation of each hormone.

**Figure 5:**
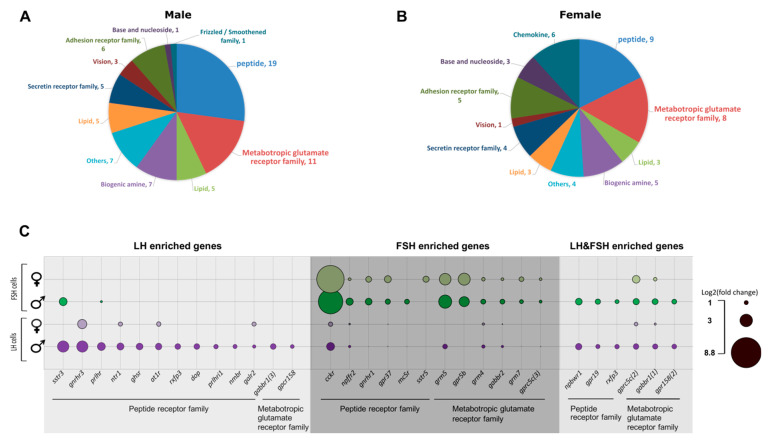
Enriched GPCRs in LH and FSH cells. (**A**,**B**) Pie charts describing the number of genes in each GPCR family that are enriched in the gonadotrophs of males (**A**) and females (**B**) according to BRIT annotation. The two GPCR families with highest number of genes are the peptide receptors family and the metabotropic glutamate receptor families. Supplementary Tables S4 and S5 contain detailed information of the genes in all identified GPCR families. (**C**) A bubble plot revealing the log2(fold change) expression of GPCRs from the peptide and metabotropic glutamate receptors families. Genes were divided to LH enriched, FSH enriched, and LH and FSH enriched genes according to the clustergram analysis (Figure 3B,C). Each row represents the fold change of genes in the FSH (green) or LH (magenta) cells compared to the negative, in males or females respectively. Though GnRH is considered the main hormone regulating gonadotropins activity, this analysis reveals a complete repertoire of hormones regulating each gonadotroph in a specific manner, and in some cases they are thought to be more significant than GnRH as their fold expression is higher, such as CCK in FSH cells and SST in LH cells. The bubble plot was prepared using Excel and visualized in Inkscape.

In our transcriptome analysis, we had identified 29 GPCRs in males that were enriched in each cell type specifically, and 6 that were enriched in both LH and FSH. In females, 22 and 23 genes are enriched in LH and FSH cells, respectively, while only 2 are enriched in both (see Supplementary Tables S4 and S5 for detailed GPCR list). The GPCRs were divided into 11 families according to BRITE annotation (Figure 5A,B). The different GPCRs revealed the direct regulation of important molecules like phoenixin-20 (through GPR173), which promotes oocyte maturation in zebrafish [79] and increases GnRH, LH, and FSH transcription in the pituitary of the spotted scat [80]); Dopamine (through drd2), which is known to inhibit LH secretion in fish [1,61], and additional molecules and peptides that relate to adhesion, vision, secretion, and more. The GPCR families with the highest number of genes in males and females are “peptide receptors” and “metabolic glutamate receptors”, which will be addressed in the following sections (Figure 5C).

Not surprisingly, one of the peptide receptors with the highest fold change in LH cells is the GnRH receptor (*gnrhr*). In FSH cells, *gnrhr* is highly expressed but is only second to *cckr* (discussed in Section 2.5). In Nile tilapia, three different types of GnRH receptors have been identified [81,82]. In the current RNA-seq, we identified cell-specific enrichment of GnRHR3 (XP_019215598.1) in LH cells (Log2(fold_change)_males/females_ = 3.7/3.1) and GnRHR1 (NP_001266689.1) in FSH cells (Log2(fold_chang)_males/females_ = 2.26/1.99). The fact that each gonadotropic cell type expresses a different GnRHR form, while both respond to the same GnRH peptide, might indicate a differential cell signaling processes regulating LH and FSH secretion in fish.

The rest of the peptide receptors enriched in LH and FSH cells relate to metabolic processes like regulation of energy balance, homeostasis, growth, and appetite. Reproduction and energy balance are two physiological processes directly linked and evolutionarily conserved [66]. As in mammals also in fish, initiation of reproduction is affected by the amount of body energy reserves and its responsiveness to diverse metabolic factors [48,64,65,66]. In mammals, different hormones were suggested to regulate the interplay between food intake and growth to reproduction: ghrelin, PYY3-36, kisspeptin, and leptin [67,83,84], all of which regulate GnRH activity in the brain. In teleosts, the regulation of different metabolic factors on GtH activity was suggested to be either by regulating GnRH neurons in the hypothalamus or by stimulating gonadotrophs directly in the pituitary gland [65], however, how the different peptide differentially regulate GtH secretion is yet to be resolved.

One of the metabolic peptides regulating both cell types that was identified by its GPCRs expression is somatostatin, where somatostatin receptor 3 (*sstr3*) is enriched in males in LH cells (log2(fold change) = 3.7) but also to a lower extent in FSH cells (log2(fold change) = 2.71) and somatostatin receptor 5 iso 3 (*sstr5*) is expressed in FSH cells in females (log2(fold change) = 2.08). In tilapia, as in other fish species, somatostatin (SST) is mainly known to downregulate GH secretion [85], but also it inhibits secretion of other pituitary hormones like prolactin, ACTH, and LH [86]. In other vertebrates, SST was mainly found to regulate the pulsatile secretion of LH; in the ewe, ICV infusion of SST abolished the pulsatile secretion of LH and dramatically decreased its plasma levels [87]. In a lactating rat, SST signaling was shown to mediate the suckling-induced suppression of pulsatile LH secretion, while central injection of SST antagonist caused a significant increase in pulsatile LH release [88]. Expression of different SST receptor types on LH and FSH cells demonstrates sexual dimorphism in the regulation of SST on GtHs.

An additional three GPCRs of metabolic peptide are common to both cell types and are expressed in males; the adropin receptor (*gpr19*, log2(fold change)_LH/FSH_ = 1.46/1.82), the neuropeptide W/neuropeptide B receptor (*npbwr1*, log2(fold change)_LH/FSH_ = 2.14/2.33), and the relaxin-3 receptor (*rxfp3*, log2(fold change)_LH/FSH_ =1.55/1.46). In mammals, these GPCRs are activated by peptides that relate to diverse physiological functions [89,90,91,92]. In fish, adropin and neuropeptide W/neuropeptide B (NPWB) were associated with GH regulation [93]; however, their regulation in the HPG axis is unknown. However, relaxin-3was previously found to increase LH secretion in the rat [94], and increased *lhβ* and *fshβ* transcription in immortalized mouse gonadotrophs (LβT2 cells) [95]. In fish, relaxin-3, together with its receptor (*rxfp3*), are expressed in the gonad and are suspected to be involved with estradiol-dependent events in follicular development [96].

The expression of the above-mentioned GPCRs suggests that both cell types are regulated by the same hormones and the differential secretion of each hormone is either by different internal signal processes unique to each cell, or by additional hormones uniquely regulating each cell type. The following sections describe metabolic hormones supposed to differentially regulate each cell type according to their cell-specific GPCR expression in our transcriptome (Figure 5C).

#### 2.6.1. Hormones Directly Regulating LH Cells

In our transcriptome, we had identified GPCRs related to energy balance expressed exclusively in LH cells. Though some of the hormones are known to affect LH secretion in mammals or fish, we reveal here that in a mono-hormonal gonadotroph system their effect can be directed only to LH cells.

Ghrelin receptor, also known as growth hormone secretagogue receptor (GHSR), was enriched in LH cells in males (log2(fold_change) = 2.3). In mammals, ghrelin was first found to increase food intake, but also demonstrated a diversity of biological actions, including promoting secretion of GH and inhibiting LH secretion [97]. Ghrelin is also known to be involved in appetite and GH secretion in fish but, as opposed to mammals, it acts as a stimulatory factor in reproduction [65]. In goldfish and carp, it was shown to increase LH transcription and secretion in vivo and in vitro using dispersed pituitary cell cultures [98,99,100].

Two different isoforms of the prolactin-releasing peptide receptor (*prlhr* and *prlhri1*; GPR10) are enriched in LH cells in males (log2(fold change) = 2.6/1.6, *prlhr* and *prlhri1* respectively). Prolactin-releasing peptide (PrRP) is known to regulate PRL secretion in the context of osmolarity adaptation in tilapia [101,102]. The involvement of PrRP along the HPG axis in fish is vague. In mammals, PrRP expression seems to be affected by gonadal estrogen secretion, where its expression increases in female rats in proestrus and after administration of estrogen or progesterone in ovariectomized rats [103], associating its activity to the reproductive cycle.

An additional homeostasis-related receptor is the angiotensin II receptor type1 (*at1r*) which is enriched in LH cells in females (Log2(fold change) = 1.6) and males (Log2(fold change) = 2.2). In mammals, angiotensin II is a part of the renin-angiotensin system known for regulating electrolytes and blood pressure, but has also been shown to regulate prolactin, ACTH, and LH secretion [104]. In fish, angiotensin II is mainly involved in blood pressure and water homeostasis [105] and the only evidence for its role in reproduction is due to the expression of angiotensin-converting-enzyme 2 (*ace2*) in the gonad of rainbow trout [106], supporting a role of at1r expression in LH cell secretion.

The Neurotensin receptor (ntr1) is enriched in LH cells in females and males (Log2(fold change)_males/females_ = 2.3/1.52). Many studies have revealed versatile physiological and behavioral roles for Neurotensin, like body temperature regulation, feeding, analgesia, ethanol sensitivity, psychosis, substance use, and pain [107,108]. Though in fish there is no direct evidence for its effect on reproduction, in mammals, Neurotensin inhibits LH secretion indirectly via GnRH neurons [107] and directly on hemi-pituitaries incubated in vitro [109]. Due to the unique expression of *ntr1* in LH cells, it is probably also regulating its secretion in fish.

The Delta opioid receptor (*dop*) is enriched in LH cells in males (Log2(fold change) = 1.9). Opioids regulate pain, behavior, and appetite, and are considered to have negative effects on LH secretion in mammals [110,111]. In fish on the other hand, opioid action on LH secretion can be contradicting and was suspected to affect the hypothalamic control of LH secretion. In carp, opioids inhibit LH secretion in females and stimulate LH secretion in males through the GnRH and dopaminergic systems [112,113]. In a Mozambican tilapia (*Oreochromis mossambicus*), the endogenous opioid peptide leu-enkephalin had stimulatory and inhibitory effects on the ovary suspected to be mediated through GnRH [114]. The opioid receptor expression on LH cells in our transcriptome analysis implies its regulation is directly on LH cells.

Galanin receptor 2 (*galr2*) is enriched in LH cells in females and males (Log2(fold change)_females/males_ = 1.49/1.2). Galanin is an important neuropeptide associated with regulation of feeding and reproductive behavior. In some fish species, projections of galanin expressing neurons were found in the proximal pars distalis [65], potentially increasing the secretion of prolactin, GH, and LH [115]. Spexin (SPX), an additional neuropeptide that was found to be a functional agonist of *galr2* [16,116], inhibited LH- and to a lesser extent FSH- secretion in vivo and directly inhibited the action potential in LH cells [16], confirming *galr2* expression in LH cells.

Neuromedin receptor B (*nmbr*) is enriched in LH cells in males (Log2(fold change) = 1.4). Neuromedin B (NMB) is a highly conserved bombesin-related peptide that affects smooth muscle contraction, satiety, thermoregulation, stress, fear, and other behavioral responses [117]. In fish, there is no evidence for its activity in the literature. In mammals, little is known regarding its activity. In the HPG axis, a recent study cloning NMB and its receptor in pigs reveals differential expression of NMB and NMBR during development and reproductive stages in the pituitary [118], connecting its activity to the HPG axis.

Our results are the first direct evidence for the local regulation of these important metabolic and homeostatic hormones on LH cells, Though most of the hormones were suspected to regulate LH activity via the hypothalamic center, their GPCRs expression in LH cells reveals how LH is directly and specifically regulated by these hormones.

#### 2.6.2. Hormones Directly Regulating FSH Cells

To our surprise, the main GPCRs identified in our transcriptome analysis that are enriched in FSH cells relate mainly to appetite, homeostasis, and especially glutamatergic and GABAergic systems. One of the novel findings is the potential direct regulation of FSH by the gastrointestinal peptide, CCK, since its GPCR (*cckr*) is the highest enriched in FSH cells (log2(fold change)_male/female_ = 7.92/8.85), we suspect that CCK might be a leading candidate for FSH regulation in fish. In mammals, different studies have revealed the existence of GnRH-independent FSH pulses [119,120,121], implying an additional unknown FSH releasing factor [122]. In fish, the direct regulation of GnRH on FSH transcription/secretion is even less characterized. In some species like Nile tilapia, Atlantic cod, and salmon, it was shown to directly stimulate FSH cell activity [9,58,123] but to a lesser extent than LH, implying there is an additional candidate regulating FSH activity.

An additional GPCR that was enriched in FSH cells in females (Log2(fold change) = 1.05) and males (Log2(fold change) = 2.42) was the Neuropeptide FF receptor 2 (*npffr2*). Belonging to the RFamide receptors family, this receptor was recently described as a regulator of diet-induced thermogenesis and bone homoeostasis [124]. RFamide neuropeptides play different roles related to pain response, energy homeostasis, anti-inflammation, and more [125]. RFamide neuropeptides were shown to inhibit GnRH activity [126] and also directly affect gonadotrophs by inducing LH and FSH secretion from fish pituitary cell culture [9], supporting npffr2 expression in FSH cells.

Two of the GPCRs that are specifically enriched in FSH cells are less known to be involved either in FSH regulation or in the HPG axis in general: the melanocortin receptor 5 (*mc5r*) and prosaposin receptor (*gpcr37*). MC5R is specifically enriched in FSH cells in males (log2(fold change) = 1.67) and is considered part of the melanocortin system. Though different melanocortins are known to regulate the interplay of hypothalamic regulation on food intake and the hormones involved in the HPG axis in mammals [127], the specific roll of MC5R was primarily associated with exocrine gland physiology [128,129] and pheromone-induced aggression in the rat [130]. In fish, MC5R activity is even less studied and proposed to be related to steroidogenesis and lipolysis [131]. Though the effects mediated by MC5R on FSH activity are unknown, its expression might imply for an exclusive activity on FSH in fish. G*pcr37* is highly enriched in FSH cells in males (log2(fold change) = 1.81) and females (log2(fold change) = 2.41). Its ligand, prosaposin (also known as SGP-1), is an intriguing multifunctional protein that plays roles both intracellularly as a regulator of lysosomal enzyme function, and extracellularly as a secreted factor [132]. The role of this receptor along the HPG axis is still unknown in vertebrates.

The metabotropic glutamate receptor family is divided into two main types of receptors: glutamate receptors and GABA receptors. While only two GPCRs from this family are enriched specifically in LH cell, seven receptors are enriched specifically in FSH cells, suggesting that FSH cells are more susceptible to the direct regulation of GABA and glutamate activity. Glutamate and GABA are both known for their activity in the central nervous system as excitatory and inhibitory neurotransmitters, respectively [133]. In mammals and fish, both neurotransmitters are proposed to increase FSH activity [7,134].

GABA type B receptor subunits (*gabbr1* and *gabbr2*) are differentially enriched in LH and FSH cells; the type1 iso3 is enriched uniquely in LH cells in males (log2(fold change) = 1.9) and the type1 iso1 is enriched in both cell types (FSH_log2(fold change)males/females_ = 2.06/1.3, LH_log2(fold change)males/females_ = 1.73/0.84), while the type 2 subunits are enriched in FSH cells (log2(fold change)_males/females_ = 1.66/1.06). Since only heterodimeric GABAB receptors, containing both subunit types, are functional [135], the higher expression of both subunits in FSH cells support our hypothesis that GABA is mainly regulating FSH. In addition, three types of glutamate metabotropic receptors were uniquely enriched in FSH cells; *grm5* (log2(fold change)_males/females_ = 4.3/3.83), *grm4* (log2(fold change)_males/females_ = 1.74/1.29), and *grm7* (log2(fold change)_males/females_ = 1.43/1.68), supporting the direct regulation of glutamate on FSH cell activity.

The GPCR genes enriched in FSH cells suggest different candidates of hormones directly regulating FSH. Whereas the high expression of *cckr* in FSH cells uncovers for the first time the importance of CCK in FSH regulation, in addition the vast repertoire of GABAergic and glutamatergic receptors reveals the significance of the GABA/glutamate system in specifically regulating FSH cells. How each of these hormones affects FSH activity, and the specific relationship between them, is yet to be resolved.

### 2.7. Enriched Genes Conserved between Mammalian and Fish Gonadotrophs

**Figure 6:**
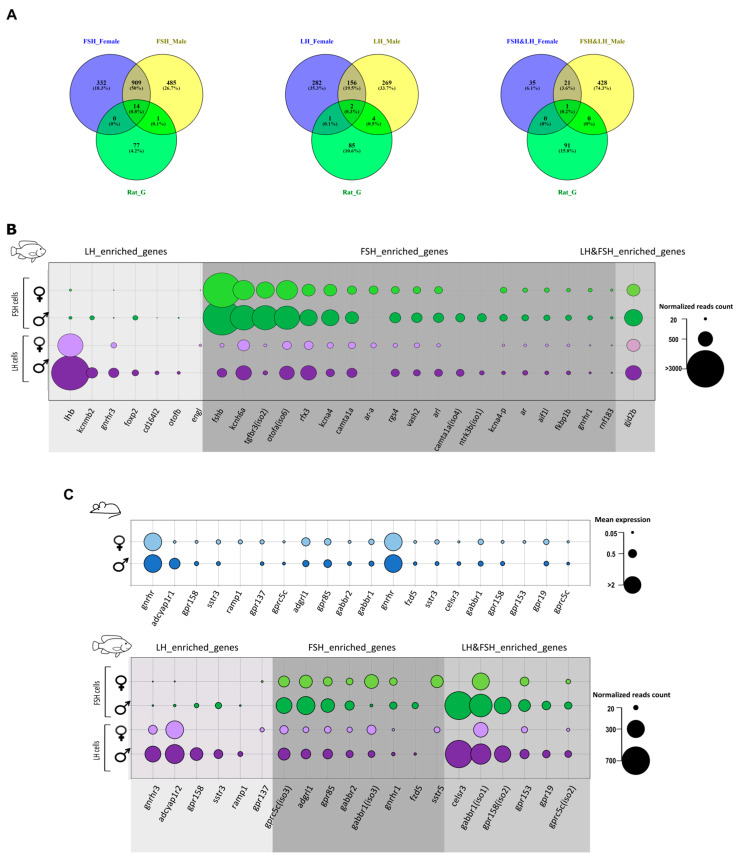
Shared genes between Nile tilapia gonadotrophs and rat gonadotrophs. (**A**)Venn diagrams describing the number of shared genes between the dominant genes expressed in rat gonadotrophs (according to Fletcher et al.) [26] and enriched genes of tilapia specific gonadotropic cell types in each sex, the classification of enriched genes in each cell type are according to the clustergram analysis (Figure 3B,C). To ensure gene ID are compatible between the different organisms, the rat IDs were converted to the zebrafish IDs using g:profiler orthology search tool and the tilapia ID to zebrafish ID as described in the methods (Section 3.7). From the 95 dominant genes identified in the rat gonadotrophs only 23 genes were common to tilapia, most of which are in FSH cells. Supplementary Table S6 lists the common genes and their expression values in each cell type. Venn diagram was created using Venny_2.1 (https://bioinfogp.cnb.csic.es/tools/venny/) (accessed on 24 September 2019). (**B**) A bubble plot describing the expression in tilapia of the shared genes between the rat gonadotrophs (males and females) and tilapia gonadotrophs according to the Venn diagrams in A. The size of the bubble reflects the normalized read (NR) count in each cell type in each sex and the genes are arranged according to their classified cluster (LH enriched, FSH enriched, of LH and FSH enriched). In some cases, the same gene in the rat contains different isoforms which are unique to each cell type in tilapia like *gnrhr* or *sstr* and in some cases different genes are classified in specific cell type like the *ar* gene in FSH cells and the *kcnmb2* gene in LH cells. This ability to classify the common genes to each cell type can give us a clue regarding the regulation of each gonadotropic hormone also in mammals. The only gene common to both cell type is the *gjd2b* gene revealing the importance of this unique gap junction in vertebrate gonadotrophs. (**C**) A bubble plot describing the expression of GPCR genes that are common to mammals (according to Fletcher et al.) [26]) and fish. The genes are arranged according to their classified cluster (LH enriched, FSH enriched, LH and FSH enriched). Bubble size represents the mean expression or the normalized reads of rat and tilapia gonadotrophs respectively in males and females. Though in the rat GnRH is the highest expressed GPCR in tilapia, other GPCRs have higher expression values in each cell type, especially in FSH cells, suggesting for different candidates regulating FSH activity. Supplementary Table S7 lists the common GPCRs and their expression values in each cell type in rat and tilapia. Bubble plots were prepared using Excel and visualized in Inkscape.

Determination of the different genomic, cellular, and endocrine factors that regulate specifically LH and FSH secretion in mammals is challenging because both hormones are secreted from the same cell. Since the mono-hormonal gonadotroph system had likely evolved from a bi-hormonal system, we suspect that conserved genes that are unique to a specific cell type in the mono-hormonal system will regulate the same hormone in the bi-hormonal system. Therefore, we compared the fish transcriptome analysis to that of the rat gonadotroph [26]. The list of genes described in this section relates to key functions that regulate gonadotrophic cell activity. Since the single cell method used for the rat adenohypophysis transcriptome analysis is essentially different from the transcriptome used in the current study, we cannot speculate regarding the non-detected genes in this context, therefore, we only compared identified genes (Figure 6). Moreover, here we highlight only the genes that are uniquely enriched in the gonadotrophs of both tilapia and rat, therefore genes that are common to other cell types are not discussed. Out of the 95 dominant genes in the rat gonadotrophs only 15 were shared with tilapia FSH cells (Figure 6A) and 7 with LH cells (Figure 6B). Out of the 95 genes, only one orthologue was expressed in both gonadotropic cell types (Figure 6C): the gap-junction delta 2 protein (*gjd2b*), which encodes connexin 36 (Cx36). Gap junctions are suggested to be important to ensure efficient, synchronous secretion of gonadotropins in mice [136] and in LH cells in Nile tilapia [17]. Whereas Cx36 has been identified to specifically mediate coupling between mammalian gonadotrophs [136], this is the first evidence for its expression in fish gonadotrophs. Cx36 expression in rat and fish gonadotrophs suggest the functional important of the coupling mechanisms in both mono- and bi-hormonal gonadotrophs. Moreover, this conserved evolutionary gene may serve as a candidate for a marker of gonadotrophs in the vertebrate pituitary.

Electric excitability of gonadotropic cells is directly related to their activity and secretion [53,58,59]. Therefore, the different ion channels expressed in each cell might suggest that there is a different excitability pattern for LH and FSH which finally affect their release patterns. Few potassium channels are conserved between rat and tilapia. In tilapia FSH cells, specific potassium voltage gated channels are enriched (*kcnh6a*, NRmales/females=1509.5/951.5, and two isoforms of *kcna4*, average NR= 295.9) while in tilapia LH cells the β subunit of a calcium activated potassium channel (*kcnmb2*, NR = 276.3) is enriched in males. KCNM channels were specifically shown to regulate Ca2+ oscillation and specifically affect LH secretion in rat gonadotrophs [54]. KCNM channels are composed of a pore-forming α subunit and additional auxiliary β and γ subunits. The auxiliary subunits can be differentially expressed in different cells [137]. The β4 subunit that is only enriched in male tilapia LH cells is also conserved in male rat gonadotrophs [26], and is known to slow down the activation and deactivation kinetics of the KCNM channel by decreasing Ca2+ sensitivity at low Ca2+ concentrations and increasing Ca2+ sensitivity at high Ca2+ concentrations [137,138], therefore contributing to the oscillatory properties of LH cells in mammals, that were suggested also in fish [55]. This unique modulation of the KCNM channel can be a conserved function that reflects different excitatory properties between LH and FSH cells in fish and mammals.

Mouse gonadotrophs express the androgen receptor (*ar*), which plays an important role in the feedback regulation from the gonads [139]. Gonadotroph-specific knockout of mouse *ar* disrupted gonadal development and fertility. In the fish pituitary, the expression of *ar* is correlated to different developmental stage of the gonad and is highly expressed in reproducing Nile tilapia [140,141,142]. Three different *ar* paralogs (*arα*, *ar like,* and *ar*) are enriched in FSH cells (average NR = 132.9), suggesting their activity is mainly related to FSH secretion during vitellogenesis. Additional transcription factors and signal transduction regulators are conserved between rat and tilapia like; *rgs4*, *foxp2*, *otofb*, and more, however, how they affect each hormone regulation is unknown.

To identify endocrine hormones regulating the activity of FSH and LH cells, we looked at the conserved GPCRs between tilapia and rat gonadotrophs (Figure 6E), and as much as 20 of the 70 identified GPCR genes enriched in tilapia gonadotrophs were shared with the rat gonadotroph transcriptome analysis. The genes were divided to LH and FSH cell groups according to their expression in the tilapia transcriptome analysis. Other than the conserved regulation of GnRH on both gonadotrophs activity, additional receptors from the peptide family were identified like somatostatin receptor and adropin receptors, emphasizing their importance in LH and FSH regulation. Moreover, the expression of GPCR genes that relate to the metabotropic glutamate receptors family like gabbr1 and gabbr2 in the rat gonadotrophs reveals conserved GABAergic regulation on those cells, probably related to FSH secretion/transcription due to their high expression in FSH cells in tilapia. The fact that many of the GPCRs already discussed in Section 2.6 are conserved in rat gonadotrophs highlight their importance in differentially regulating gonadotroph activity in mammals.

In LH cells, pituitary adenylate cyclase- activating polypeptide type 1 receptor isoform 1 (*adcyap1r1*) is enriched in males (NR = 324.6) and females (NR = 282.7). Classified in the secretion receptor family according to BRITE analysis (see Supplementary Tables S4 and S5), this receptor and its ligand (PACAP) are known to play an important role in regulating the HPG axis in both mammals and fish [47,143,144,145]. In mammals, PACAP can either inhibit or elicit gonadotropin secretion depending on the sex, the reproductive stage, and application method [143]. In fish, PACAP increased LH secretion in the gonadotrophs of the goldfish [144], while in previous studies performed on cell culture of tilapia pituitaries, it increased both LH and FSH subunit synthesis [47]; unfortunately how it affects the secretion of FSH in fish is yet unknown. However, the specific expression of adcyap1r1 in tilapia LH cells strengthens its importance for LH activity.

Latrophilin1 (*adgrl1*) is a member of the adhesion receptor family and is found to be conserved in rat gonadotrophs and tilapia FSH cells. The activity of adgrl1 has been extensively studied as a component of black widow spider venom and was found to elicit spontaneous exocytosis of neurotransmitters from neurons and peptide hormones of endocrine cells [146]. However, this is the first evidence for its expression in FSH cells and the pituitary of fish in general. Since its expression in LH cells is relatively low, it might be a candidate for the regulation of the different secretion patterns of LH and FSH from gonadotrophs in vertebrates.

When looking at the endocrine control of gonadotropin secretion from an evolutionary perspective, few key genes which are highly conserved between very distant species had been highlighted. GnRH is considered the main neuropeptide regulating LH and FSH secretion, and even though GnRHR is the highest expressed GPCR in rat gonadotrophs, the two different isoforms of tilapia GnRHRs are not the highest GPCRs expressed in tilapia gonadotrophs. Interestingly, highly expressed GPCRs that were identified in fish transcriptome, and have higher NR counts than the GnRH receptors in fish, were not detected in the rat transcriptome, like *cckr*, *nmbr*, *ntr1*, and *drd4*. Though speculating on genes that are missing from different transcriptome analyses is problematic, the fact that these genes are highly expressed in fish, and are missing in rat, might suggest a unique adaptation for LH and FSH cells in fish that might also relate to the unique adaptation of mono-hormonal system.

## 3. Materials and Methods

### 3.1. Fish Care and Maintenance

Nile tilapia (*Oreochromis niloticus*, Lake Manzala strain) were kept and bred in a recirculating water system at 26–28 °C and fed twice daily with commercial pellets (47% protein, 6% fat, Raanan Shivuk, Israel). The double-labeled fish used for this study were grown together in a single 500-L tank. In each experiment, the fish were sampled randomly from the tank. Fish sex, weight, and gonado-somatic index (gonadal mass as a proportion of the total body weight; GSI) are detailed for every experiment; all the fish used in this study were sexually mature while their GSI ranged between 0.001–5.7% for females and 0.007–1.02% for males (Supplementary Figure S1). Double-labeled transgenic fish expressing GFP and RFP in GtH cells were the offspring of single-labeled parents; FSH:GFP that were previously described [17,18] and LH:RFP that were created as previously described [45]. Using the LR Three-way Multisite Gateway reaction (Invitrogen, Carlsbad, CA, USA) the resulting construct drove tagRFP expression in LH cells and tagRFP expression in the heart. After breeding of the single-labeled fish, the embryos were screened for red and green signals in the heart, only embryos expressing both signals were considered double-labeled fish.

All experiments were conducted in accordance with the Animal Care and Use Guidelines of the Hebrew University and were approved by the National Research Council for Care and Use of Laboratory Animals.

### 3.2. Confocal Imaging

Pituitaries of adult double-labeled Nile tilapia (FSH:GFP&LH:RFP) were fixed in 4% PFA for 2 h at room temperature and then cryoprotected by immersion in PBS containing 20% (*w*/*v*) sucrose and 30% OCT (*w*/*v*) overnight at 4 °C. Tissue was frozen in OCT blocks and sectioned in a cryostat at a thickness of 12 μm. The slides were stained with DAPI and mounted in anti-fade solution, pituitaries were imaged for RFP- and GFP-labeled cells using confocal fluorescent microscope (Leica microsystems), and images were produced using an ×20 and ×60 objectives and processed using Fiji program [147].

### 3.3. Isolation of LH and FSH Cells from Double Labeled Pituitaries

For the isolation of LH and FSH cells, pituitaries from 20 double-labeled mature males or 20 mature females of *O.niloticus* were harvested and validated using fluorescent microscopy for GFP- and RFP-labeled cells. Positive pituitaries expressing RFP and GFP from each sex were validated in a fluorescent microscope and pooled together into a modified HBS (HEPES Buffered Saline; 145 mM NaCl, 5.4 mM KCl, 1.8 nM CaCl2, 1 mM MgCl2, 20 mM D-Glucose, 20 mM HEPES). Next, the pituitaries were digested by trypsin into a single cell suspension according to [9,148]. The cell suspension was filtered through a 40μm sieve and suspended in PBS containing 30% Accumax (SCR006 Accumax cell detachment solution, Mercury-ltd, Rosh-Ha’ayin, Israel.). The procedure was repeated four times for each sex, generating four biological replicates for each sex, totaling 76 female fish (body weight 78.82 ± 2.61g; GSI 1.9 ± 0.2%) and 77 male fish (body weight 143.16 ± 7.05 g; GSI 0.22 ± 0.04%) were used (Supplementary Figure S1). The cell suspensions were sorted in a FACSAriaIII sorter using a 488 nm and 561 nm lasers to excite the GFP- and RFP-labeled cells, respectively. To determine the correct parameters of gaiting for the positively-labeled cell population (Supplementary Figure S2), different pituitaries served as controls: non-labeled, single-labeled of FSH:GFP, and single-labeled of LH:RFP. Cell viability was also determined using the fluorescent exclusion dye, propidium iodide (Merck KGaA, Darmstadt, Germany). Four fractions were collected: GFP-positive fraction enriched in FSH cells, RFP-positive fraction enriched in LH cells, GFP- and RFP-positive that contain both cell types probably due to doublets of LH and FSH cells, and negative cells fraction that contain non-LH and non-FSH pituitary cells. For each cell suspension, 23,000 to 60,000 cells were collected simultaneously.

### 3.4. RNA-Seq Library Preparation of FACS Cells

After sorting, cells were immediately centrifuged (300 × g for 4 min) and suspended in Trizol^®^ Reagent (Thermo Fisher Scientific, Waltham, MA, USA) for total RNA extraction, according to the manufacturer’s instruction. Total mRNA from each sample was dissolved in 20 μL DEPC water with concentrations ranging from 0.9 to 4.3 ng/μL (Figure 2C). RNA quality and quantity were verified using a 2200 TapeStation (Agilent Technologies). Total RNA samples were sent to the Interdepartmental Equipment Unit, Faculty of Medicine (The Hebrew University) for RNA-seq library preparation and sequencing. RNA libraries were prepared using the SMARTer^®^ Stranded Total RNA-Seq Kit v2- Pico Input Mammalian (Takara Bio, Mountain View, CA, USA which is adaptable for low quality RNA samples. The libraries were subjected to next generation sequencing using Illumina^®^ NextSeq^®^ 500 system (Illumina, Inc, San Diego, CA, USA), performing 76 bp single-end read sequencing of the 32 multiplexed samples. Each library contained at least 24 M reads (in average 45.5 M and 27.5 M for males and females, respectively).

### 3.5. RNA-Seq Library Processing, Mapping, and Annotation

Raw reads (Fastq files) were inspected for quality issues with FastQC v0.11.4 (http://www.bioinformatics.babraham.ac.uk/projects/fastqc/) (accessed on 1 September 2019) and trimmed for quality and adaptor removal using Trim Galore (https://github.com/FelixKrueger/TrimGalore) (accessed on 1 September 2019) default settings. Single end reads were mapped to O. niloticus (assembly O_niloticus_UMD_NMBU GCA_001858045.3), using STAR v 2.201 [149]. Mapped reads were processed to remove identified contamination of ribosomal and bacterial RNA. From the uniquely mapped reads, an average of 43% and 47% were assigned to O. niloticus known genes in males and females, respectively. Finally, the average number of reads that were assigned to a known gene in the O. niloticus genome, for each cell type (thus constituting the data on which the analysis is based), were, in the male pituitary FACS cells: LH cells; 2.3 M reads, FSH cells; 4.2 M reads, negative cells; 2.8 M, “before sorting” control cells; 4.5 M reads. For the female pituitary FACS cells, they were LH cells; 1.6 M, FSH cells; 1.5 M reads, negative cells; 1.4 M reads and the “before sorting” cells; 3 M reads per sample. FASTQ files and the results of DESeq analysis discussed in this study are available on the National Center for Biotechnology Information (NCBI) Gene Expression Omnibus (GEO) [150,151] through accession number GSE159470.

### 3.6. Identifying Uniquely Expressed Genes in LH and FSH Cells

Differential expression analysis was performed using DESeq2 R package. In the FACS sorted cells, each LH or FSH library was analyzed against the negative library. More than 3K genes with normalized read counts greater than 100 were identified. In total, 10,713 and 10,134 genes were differentially expressed (FDR corrected *p* < 0.05) between the gonadotrophs and the negative fractions in males and females, respectively. The distribution of the number of genes with the different read counts in each library is detailed in Supplementary Table S8. Volcano plots describing the differentially expressed genes (log2 > 1, *p* < 0.05) is detailed in Supplementary Figure S3.

To decide the fold change cutoff of enriched genes (EGs) for LH and FSH cells, we looked at the RNA-seq expression of common pituitary hormones in the different FACS fractions (Figure 3A). In order to increase the reliability of the results, only genes with a log2 fold change greater than one (above the red line in Figure 3A) were considered EGs in LH and FSH samples, eliminating genes with low expression that might arise from unwanted cell contamination in the sorted fractions. Therefore, EGs were selected according to the following cutoff criteria: FDR corrected p-value lower than 0.05 and absolute fold change expression higher than 2. To create gene lists for uniquely expressed genes in LH, FSH, or both cell types, we performed a hierarchical clustering analysis using MATLAB (MATLAB_R2107a) based on the filtered EGs, using a Seuclidean distance matrix and a complete linkage method for the cluster analysis.

### 3.7. Functional Analysis of Enriched Genes in FSH and LH Cells

The well annotated zebrafish (*D. rerio*) gene homologs were used as a reference for further functional analyses of the EGs. For the purpose of assigning zebrafish homologs to tilapia protein sequences, they were further aligned against a database containing all zebrafish protein sequences (genome version GRCz11) using GHOSTX—An Improved Sequence Homology Search Algorithm [152]. Sequences were selected as homologues if BLAST e-value were lower than 10–5 and sequence identity was higher than 60%. The zebrafish proteins with the highest blast bit-score (along with its annotations) were assigned to each one of the tilapia proteins as their representative homolog. Enrichment analysis of GO terms and KEGG pathway was performed using the g:Profiler [153], the genes were categorized according to their fold change in each cell type, and uploaded to g:Profiler using the zebrafish organism as reference. The different enriched GO annotations were later grouped using the CateGOrizer tool (https://www.animalgenome.org/tools/catego/) (accessed on 1 September 2019).

### 3.8. Real-Time PCR Validations

cDNA preparation was accomplished with 2 ng of the FACS cells mRNA using the Verso cDNA Synthesis Kit (Thermo Fisher Scientific, Waltham, MA, USA) according to the manufacturer’s instruction. Real-time PCR validations were performed as described previously [154]. cDNA (3 μL) was used as template in a 20 μL final reaction volume with Platinum SYBR Green qPCR SuperMix (Invitrogen). Each set of primers were validated first for specificity and efficiency using 6 dilutions (×5) of pituitary cDNA. To assess the mRNA expression of lhβ, fshβ, and gh in each fraction, mRNA levels (the averaged of two technical replicates) were normalized against the reference gene, EF1α, using the comparative threshold cycle method (−∆CT). Ef1α was previously shown to be a suitable reference gene for gene expression in several fish species including tilapia [14,154]. Primer sequences are detailed in Supplementary Table S1.

### 3.9. Comparing Tilapia and Rat Transcriptome Analysis

Gene lists from the rat single cell transcriptome analysis were published and kindly provided by Dr. Stojilkovic (National Institutes of Health) [26]. We obtained two different lists, one for the significantly dominant genes expressed in rat gonadotrophs and a second matching our specific GPCRs list obtained by BRITE analysis [155] and are expressed in at least 5% of the gonadotroph identified in the single cell analysis. Tilapia gene IDs were converted to rat IDs using g:profiler orthology search tool. The common genes between rat gonadotrophs to tilapia LH and FSH cells were identified using a Venn diagram (Venny 2.1).

## 4. Conclusions

By successfully separating LH and FSH cells from the pituitaries of Nile tilapia, we had identified cell-specific and sex-specific enriched genes that may contribute to different excitatory mechanisms, different signal transduction properties, and different hormonal regulation of these cells. We identified different types of GnRH receptors that are differentially expressed in each cell type, highlighted conserved peptides and metabotropic glutamate receptors that might contribute to the differential secretion and synthesis of each of the GtHs and potentially link between reproduction and other physiological mechanisms. One novel finding in this study was the high levels of *cckr* expression in FSH cells, which we hypothesize to be a link between the metabolic and reproductive status of the fish. We had identified conserved orthologues by comparing our enriched genes to that of the rat gonadotroph, revealing their contribution to the differential regulation of LH and FSH, and emphasizing their importance in gonadotroph activities.

## Figures and Tables

**Table 1:** Top 10 upregulated genes according to fold change in FACS sorted cells. Genes were grouped according to suggested function classification. Each gene is one of the top 10 genes in one or more of the different FACS samples as mentioned in the “TOP 10” column. Some of the genes are also enriched in additional samples but not included in their top 10 genes as mentioned in the “upregulated also in” column. See Supplementary Table S3 for fold change and expression values of each gene in each fraction.

	Name	Protein Name	TOP 10	Up Regulated Also In
**Gonadotrophs secretion**	*fshβ*	follitropin subunit beta isoform X1	FSH(M&F)	non
	*lhβ*	gonadotropin subunit beta-2	LH(M&F)	non
	*esr2*	estrogen receptor beta isoform X2	LH&FSH(M&F)	
	*pgr*	progesterone receptor	LH(F)	LH(M)
**Neuroactive ligand receptor interaction**	*cckr*	cholecystokinin receptor	FSH(M&F)	LH (M&F)
	*apln*	apelin	LH(M)	FSH(M) LH&FSH(F)
**Cell signaling**	*pdeh6h*	retinal cone rhodopsin-sensitive cGMP 3\′,5\′-cyclic phosphodiesterase subunit gamma	FSH(M&F)	
	*pcp4*	calmodulin regulator protein PCP4 isoform X3	LH(M) FSH(F)	FSH(M) LH(F)
**Cell morphology**	*xirp2*	xin actin-binding repeat-containing protein 2 isoform X1	FSH(F)	FSH(M) LH(F)
	*thbs4b*	thrombospondin-4-B	LH(M&F)	
	*col5a2*	collagen alpha-2(V) chain	LH(F)	FSH(F) LH(M)
	*cdh16*	cadherin-16	FSH(F)	FSH(M)
**Post translation modification**	*ndst4*	bifunctional heparan sulfate N-deacetylase/N-sulfotransferase 4 isoform X1	LH(M)	FSH(M&F) LH(F)
	*dclk3*	serine/threonine-protein kinase DCLK3 isoform X1	LH(M)	FSH(M) LH&FSH(F)
	*pcdstpk*	probable cyclin-dependent serine/threonine-protein kinase DDB_G0292550	LH(M&F)	
	*tyr*	tyrosinase	FSH(M)	
**Protein trafficking**	*tnfaip2*	tumor necrosis factor alpha-induced protein 2	FSH(M&F)	
	*tnfaip2*	tumor necrosis factor alpha-induced protein 2	FSH(M&F)	
	*tnfaip2*	tumor necrosis factor alpha-induced protein 2	FSH(M)	FSH(F)
**Cell differentiation**	*fgfr4*	fibroblast growth factor receptor 4	LH(M)	LH(F)
**Transcription factore**	*zax*	homeobox protein zampogna	FSH(M)	LH(M&F) FSH(F)
	*hnf4a*	hepatocyte nuclear factor 4-alpha isoform X1	LH(F)	FSH(F) LH&FSH(M)
	*esrrb*	LOW QUALITY PROTEIN: steroid hormone receptor ERR2	FSH(F)	LH (F) LH&FSH(M)
**Chemokine receptore**	*xcr1*	chemokine XC receptor 1-like isoform X1	LH(F)	
**Other**	*ucp*	uncharacterized protein LOC102076330	FSH(M)	LH(M) LH&FSH(F)
	*ucp*	uncharacterized protein LOC102077685 isoform X1	LH(M&F)	FSH(M)
	*f6i1*	fucolectin-6 isoform X1	LH(F)	

## Data Availability

FASTQ files and the results of DESeq analysis discussed in this study are available on the National Center for Biotechnology Information (NCBI) Gene Expression Omnibus (GEO) [150,151] through accession number GSE159470.

## Supplementary Materials

The following are available online at https://www.mdpi.com/article/10.3390/ijms22126478/s1.

## Abbreviations

Gonadotropin-releasing hormone	GnRH
Luteinizing-hormone	LH
Follicle-stimulating-hormone	FSH
Gonadotropin hormones	GtHs
Hypothalamus-pituitary-gonad	HPG
Rostral pars distalis	RPD
Proximal pars distalis	PPD
Pars intermedia	PI
Pars nervosa	PN
LHβ subunit	lhβ
FSHβ subunit	shβ
Growth hormone	gh
Prolactin	prl
Thyroid-stimulating hormone	tshβ
Somatolactin	sl
Pro-opiomelanocortin	pomc
Glycoprotein α-subunit	gcα
Normalized read	NR
Enriched genes	EG
Gene ontology	GO
Neurokinin B	NKB
Estrogen receptor 2	esr2
Progesterone receptor	pgr
17α-20β-Dihydroprogesterone	DHP
Estradiol	E2
Somatostatin receptor	sstr
Somatostatin	SST
Adropin receptor	Gpr19
Neuropeptide W/neuropeptide B	NPWB
Relaxin-3 receptor	rxfp3
Secretagogue receptor	ghsr
Prolactin-releasing peptide receptor	prlhr, gpr10
Prolactin-releasing peptide	PrRP
Angiotensin II receptor type1	at1r
Angiotensin-converting-enzyme 2	ace2
Neurotensin receptor	ntr1
Delta opioid receptor	dop
Galanin receptor 2	galr2
Spexin	SPX
Neuromedin receptor B	nmbr
Neuromedin B	NMB
Cholecystokinin	CCK
Apelin	apln
Calmodulin regulator protein or PEP-19	pcp4
Phosphodiesterase 6H	pde6h
Calmodulin-dependent kinase II	CaMKII
G-protein coupled receptor	GPCR, gpr
Neuropeptide FF receptor 2	npffr2
Melanocortin receptor 5	mc5r
Prosaposin receptor	gpcr37
Gamma-Aminobutyric acid	GABA
GABA type B receptor subunit1	gabbr1
GABA type B receptor subunit	gabbr2
Gap-junction delta 2 protein	gjd2b
Connexin 36	Cx36
Potassium voltage-gated channel, subfamily H	eag-related
member 6a	kcnh6a
Potassium voltage-gated channel subfamily A member 4	kcna4
Calcium-activated potassium channel subunit beta-2	kcnmb2
Androgen receptor	ar
G protein signaling	rgs
Adenylate cyclase-activating polypeptide type 1 receptor isoform 1	adcyap1r1
Latrophilin1	adgrl1
Dopamine receptor	drd
