# Supplementary material for: Differential Regulation of Gonadotropins as Revealed by Transcriptomes of Distinct LH and FSH Cells of Fish Pituitary
Source: Int J Mol Sci. 2021 Jun 17;22(12):6478. doi: 10.3390/ijms22126478 (PMC8234412; doi:10.3390/ijms22126478)
Supplement: Supplementary file 1 [file ijms-22-06478-s001.zip › ijms-1221350-supplementary.pdf]

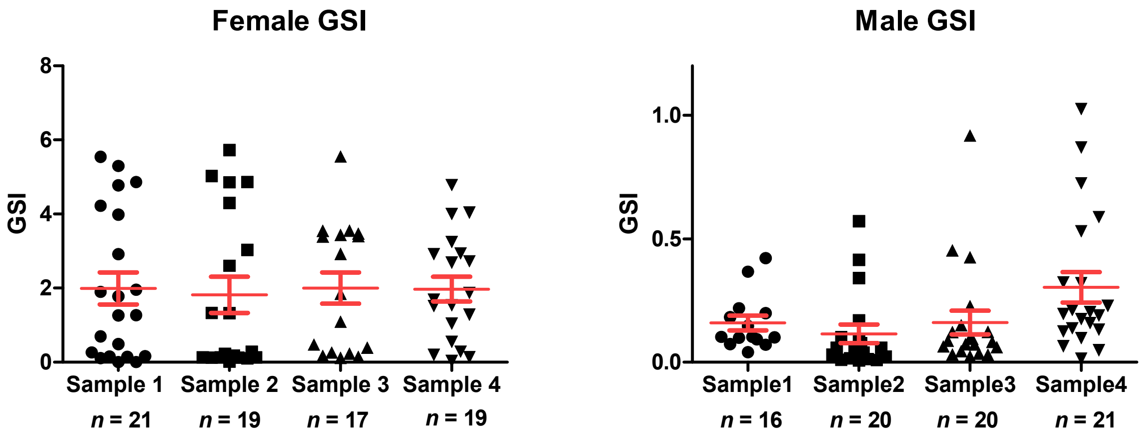

Figure S1. Gonado somatic index (gonad weight/total weight) of the fish used for pituitary harvesting in each FACS sample.

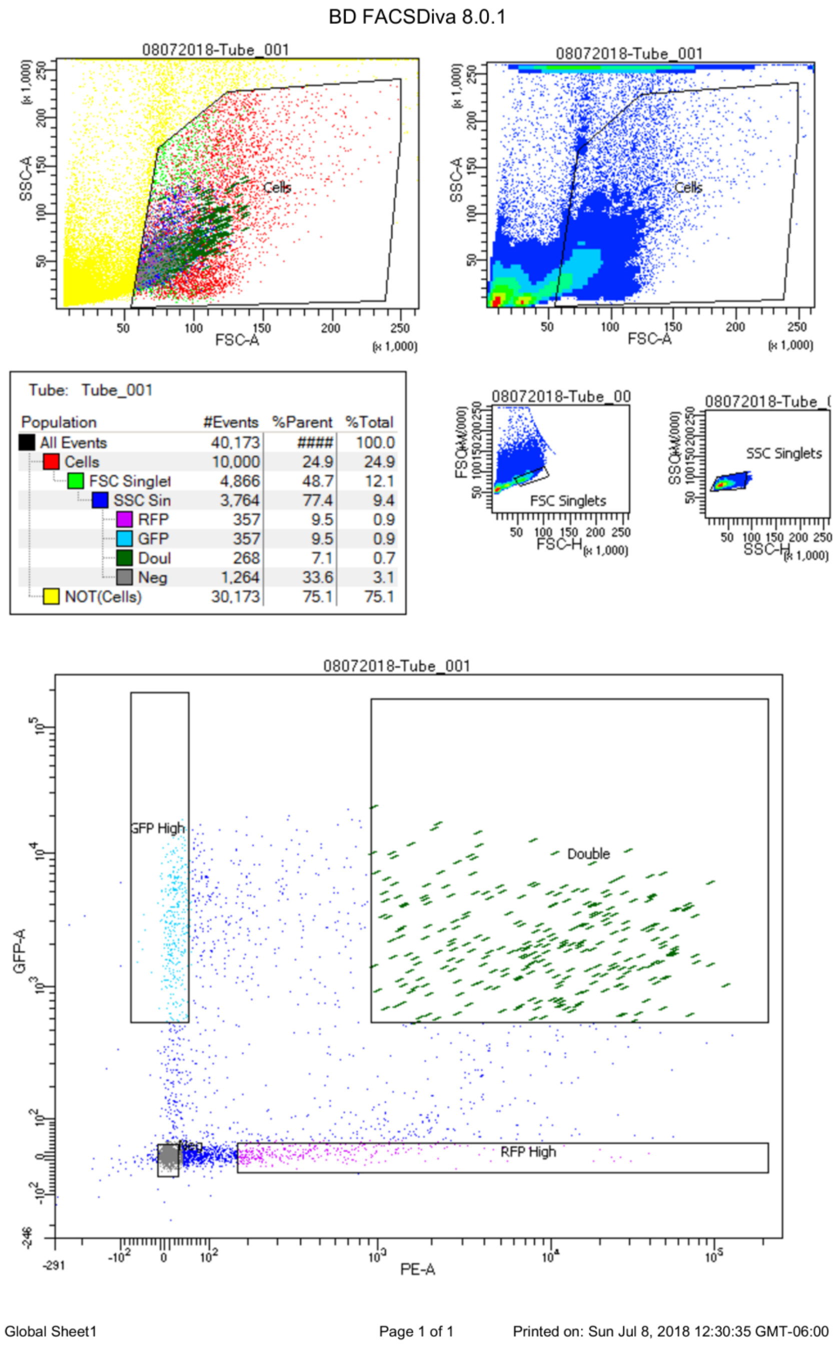

Figure S2. The complete gating parameters of the FACS assay for LH and FSH cells separation.

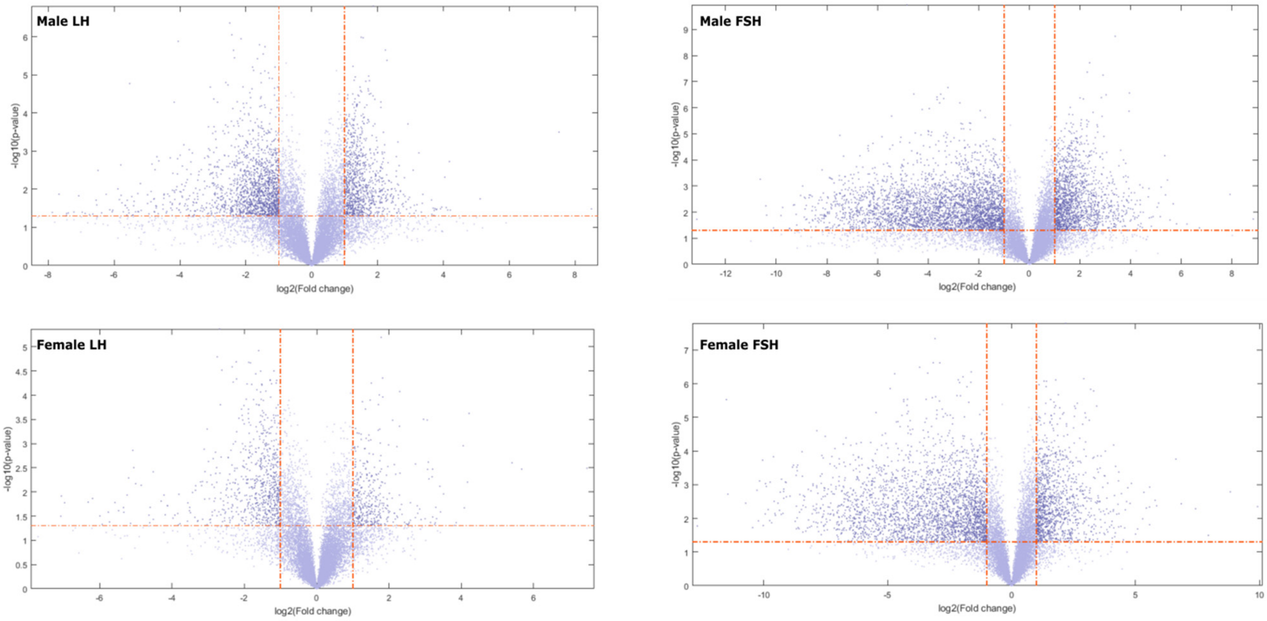

Figure S3. Volcano plots of the genes in each RNAseq library. with read count >20. The plots describing the gene distribution in each fraction according to their fold change and p-value compared to the negative fraction. Red lines represent the cutoff of genes with $p < 0.05$ and fold change >2. Volcano plots were prepared using MATLAB mavolcanoplot command.

Table S1. Primers used for real time validation of the sorted fractions.

Name	Position	5′ to 3′ sequence	Slope	R^2
LH_F	109	TATCTCCTGCAGCGGCCTT	−3.35	1
LH_R	327	TACACCAGGTGGGCAATCG		
FSH_F	45	AGAGGATGCAGCTGGTTG	−3.52	1
FSH_R	171	GTTGGGATCCTTCTGGAAAG		
GH_F	151	TGCTCGCCCAGAGACTCTTC	−3.52	1
GH_R	135	TGGGAAACTCCCAGGACTCA		
EF1A_F	438	GCACGCTCTGCTGGCCTTT	−3.49	1.93
EF1A_R	688	GCGCTCAATCTTCCATCCC		

Table S2. $\log_2$(fold change) values of pituitary hormones in the collected cell fractions.

Protein Name	Gene ID	FSH Cells Males	FSH Cells Females	LH Cells Males	LH Cells Females
fshβ	fshb	8.837754463	9.905770131	0.868759321	0.080738747
lhβ	LOC100534501	0.393589203	0.897771633	8.502444908	7.598570358
cgα	cga	4.407142408	3.732029127	2.424932231	0.430062612

gh	LOC100534452	−5.921503739	−7.805671863	−3.248507567	−3.573404686
prl(1)	LOC100534522	−4.212938494	−6.043186502	−2.711944967	−2.833833834
prl(2)	LOC100534523	−4.984764177	−6.563761933	−3.066079508	−3.516573681
tshβ	LOC100534562	−7.405845506	−12.05531371	−2.110557386	−2.69508372
pomc	pomc	−6.645254836	−7.413334818	0.877120029	0.69733075
sl	LOC100711096	−5.577935741	−8.79004548	0.887309161	0.057638845

Table S3. Top 10 genes expressed in each fraction according to the fold change in respect to the negative fraction.

					Log2Fold-Change		**Padj**		**Av_Norm**	
	**Row.Names**	**GeneID**	**Protein Product**	**Protein Name**	LH.VS.Neg	FSH.VS.Neg	**LH.VS.Neg**	**FSH.VS.Neg**	**FSH**	**LH**
**LH Male**	LOC100534501	100534501	XP_025753499.1	gonadotropin subunit beta-2	8.50	0.39	4.95×10^{-38}	6.46×10^{-01}	20.29	5653.06
	esr2	100534556	XP_025756490.1	estrogen receptor beta isoform X2	5.86	5.41	2.60×10^{-34}	4.49×10^{-31}	399.39	544.83
	LOC100703182	100703182	XP_019219020.1	calmodulin regulator protein PCP4 isoform X3	5.24	3.90	2.66×10^{-13}	3.70×10^{-08}	42.01	107.30
	apln	106098248	XP_013126785.1	apelin	5.05	4.32	2.75×10^{-23}	3.42×10^{-18}	112.60	187.34
	LOC100692465	100692465	XP_003451467.2	fibroblast growth factor receptor 4	5.00	−0.52	1.87×10^{-28}	3.82×10^{-01}	11.18	524.88
	LOC100693277	100693277	XP_003451807.1	thrombospondin-4-B	4.97	−0.47	6.05×10^{-13}	6.42×10^{-01}	2.24	115.14
	dclk3	100698746	XP_005478670.1	serine/threonine-protein kinase DCLK3 isoform X1	4.72	3.87	6.38×10^{-14}	3.39×10^{-10}	117.65	214.06
	LOC102077685	102077685	XP_025752955.1	uncharacterized protein LOC102077685 isoform X1	4.54	1.45	3.32×10^{-15}	2.41×10^{-02}	20.21	177.06
	LOC100695867	100695867	XP_003454281.2	bifunctional heparan sulfate N-deacetylase/N-sulfotransferase 4 isoform X1	4.39	5.10	1.09×10^{-24}	3.69×10^{-35}	1191.13	728.43
	LOC102083321	102083321	XP_019219833.1	probable cyclin-dependent serine/threonine-protein kinase DDB_G0292550	4.24	−1.93	1.11×10^{-09}	1.00×10^{-01}	0.54	53.01
**FSH Male**	fshb	100534500	XP_013130155.1	follitropin subunit beta isoform X1	0.87	8.84	3.13×10^{-01}	5.64×10^{-50}	48573.70	193.54
	LOC100690909	100690909	XP_003443146.1	retinal cone rhodopsin-sensitive cGMP 3\',5\'-cyclic phosphodiesterase subunit gamma	1.57	8.01	1.39×10^{-01}	3.85×10^{-24}	6314.32	72.42
	LOC100700667	100700667	XP_005457923.1	cholecystokinin receptor	2.62	7.92	1.05×10^{-16}	1.31×10^{-159}	20112.98	511.82
	tyr	100707700	XP_003441635.1	tyrosinase	0.95	6.35	7.14×10^{-01}	3.73×10^{-07}	28.20	0.66
	LOC100693617	100693617	XP_019204240.1	tumor necrosis factor alpha-induced protein 2	0.29	6.20	7.84×10^{-01}	4.24×10^{-29}	534.15	8.80
	LOC109195942	109195942	XP_019204245.1	tumor necrosis factor alpha-induced protein 2	−0.26	5.73	8.88×10^{-01}	1.26×10^{-14}	101.38	1.50
	LOC102076330	102076330	XP_005476240.1	uncharacterized protein LOC102076330	3.17	5.67	3.10×10^{-11}	2.26×10^{-37}	758.63	134.79
	LOC109195943	109195943	XP_019204247.1	tumor necrosis factor alpha-induced protein 2	0.30	5.50	8.81×10^{-01}	1.06×10^{-07}	127.80	3.43
	esr2	100534556	XP_025756490.1	estrogen receptor beta isoform X2	5.86	5.41	2.60×10^{-34}	4.49×10^{-31}	399.39	544.83
	LOC100705614	100705614	XP_003452020.1	homeobox protein zampogna	4.21	5.36	6.00×10^{-09}	1.71×10^{-15}	79.76	36.24
**LH Female**	LOC100534501	100534501	XP_025753499.1	gonadotropin subunit beta-2	7.60	0.90	6.31×10^{-83}	9.76×10^{-02}	12.76	1334.85
	LOC100707060	100707060	XP_019207775.1	collagen alpha-2(V) chain	5.44	2.71	7.47×10^{-17}	8.38×10^{-05}	19.28	128.33

	LOC102083321	102083321	XP_019219833.1	probable cyclin-dependent serine/threonine-protein kinase DDB_G0292550	4.47	−1.38	9.15×10^{-05}	4.62×10^{-01}	0.23	19.80
	LOC100693277	100693277	XP_003451807.1	thrombospondin-4-B	4.46	0.52	2.74×10^{-09}	6.47×10^{-01}	2.09	38.79
	esr2	100534556	XP_025756490.1	estrogen receptor beta isoform X2	4.35	5.71	1.52×10^{-20}	4.68×10^{-38}	216.91	84.90
	hnf4a	100706245	XP_005477971.2	hepatocyte nuclear factor 4-alpha isoform X1	4.12	4.60	1.64×10^{-09}	4.40×10^{-13}	54.65	40.21
	pgr	100534498	XP_005455130.1	progesterone receptor	3.63	-2.80	6.82×10^{-05}	6.93×10^{-02}	0.00	17.90
	LOC102077685	102077685	XP_025752955.1	uncharacterized protein LOC102077685 isoform X1	3.54	0.88	8.85×10^{-11}	2.13×10^{-01}	6.60	43.63
	LOC100711803	100711803	XP_025756324.1	chemokine XC receptor 1-like isoform X1	3.49	−3.82	1.71×10^{-06}	5.60×10^{-03}	0.00	33.71
	LOC100692815	100692815	XP_003450308.1	fucolectin-6 isoform X1	3.44	−3.06	4.26×10^{-04}	4.93×10^{-02}	0.00	19.50
**FSH Female**	fshb	100534500	XP_013130155.1	follitropin subunit beta isoform X1	0.08	9.91	9.53×10^{-01}	1.61×10^{-203}	33947.74	37.09
	LOC100700667	100700667	XP_005457923.1	cholecystokinin receptor	1.56	8.85	6.91×10^{-05}	7.88×10^{-174}	8826.10	55.43
	LOC100690909	100690909	XP_003443146.1	retinal cone rhodopsin-sensitive cGMP 3\',5\'-cyclic phosphodiesterase subunit gamma	0.32	7.93	7.78×10^{-01}	3.16×10^{-97}	3994.39	20.62
	LOC109195943	109195943	XP_019204247.1	tumor necrosis factor alpha-induced protein 2	1.44	7.32	5.77×10^{-01}	2.56×10^{-11}	94.40	2.09
	LOC100693617	100693617	XP_019204240.1	tumor necrosis factor alpha-induced protein 2	−0.57	6.96	7.88×10^{-01}	2.70×10^{-37}	326.99	1.42
	LOC100698925	100698925	XP_013124338.1	xin actin-binding repeat-containing protein 2 isoform X1	1.50	6.34	4.64×10^{-03}	2.08×10^{-53}	1483.47	52.59
	cdh16	100709162	XP_003445808.2	cadherin-16	0.80	6.02	7.83×10^{-01}	1.38×10^{-10}	55.52	1.39
	LOC100690064	100690064	XP_005460328.2	LOW QUALITY PROTEIN: steroid hormone receptor ERR2	1.38	5.74	7.74×10^{-02}	6.07×10^{-31}	174.38	9.35
	esr2	100534556	XP_025756490.1	estrogen receptor beta isoform X2	4.35	5.71	1.52×10^{-20}	4.68×10^{-38}	216.91	84.90
	LOC100703182	100703182	XP_019219020.1	calmodulin regulator protein PCP4 isoform X3	5.80	5.59	1.21×10^{-05}	6.07×10^{-06}	9.82	12.79

Table S4. GPCRs identified in BRITE functional analysis in males.

	BRITE functional hierarchies	Gene ID	Protein Name	log2FoldChange		Av_Norm		Padj	
				LH.VS.neg	FSH.vs.neg	LH	FSH	LH.VS.Neg	FSH.VS.Neg
**LH**	Adhesion receptor family	LOC102082324	adhesion G-protein coupled receptor D2 isoform X5	2.07	1.47	31.54	19.67	5.09×10^{-04}	1.21×10^{-02}
	Others	cnr1	cannabinoid receptor 1	1.42	−0.40	24.66	7.21	4.91×10^{-02}	6.00×10^{-01}
	Others	LOC100692145	G-protein coupled receptor 61	3.22	2.38	17.98	9.96	2.17×10^{-04}	5.76×10^{-03}
	Biogenic amine	drd2	D(2) dopamine receptor isoform X1	3.24	1.79	72.86	26.24	1.75×10^{-14}	4.37×10^{-05}
	Biogenic amine	drd3	D(3) dopamine receptor isoform X1	1.65	−3.19	26.24	0.77	2.21×10^{-02}	1.05×10^{-03}
	Biogenic amine	LOC100700659	histamine H2 receptor	1.56	−4.16	19.97	0.20	9.06×10^{-02}	1.17×10^{-03}
	Biogenic amine	LOC100534567	muscarinic receptor 4 subtype isoform X1	2.56	−0.50	50.32	6.10	2.31×10^{-10}	3.62×10^{-01}
	Lipid	gpr34	probable G-protein coupled receptor 34	1.69	−1.51	36.81	4.00	3.35×10^{-02}	6.58×10^{-02}
	Lipid	LOC102080433	prostaglandin E2 receptor EP1 subtype isoform X2	1.30	−6.10	137.53	1.16	1.71×10^{-03}	1.61×10^{-13}
	Lipid	s1pr1	sphingosine 1-phosphate receptor 1	1.02	−3.76	48.02	1.80	2.11×10^{-02}	1.88×10^{-09}

	Metabotropic glutamate receptor family	gabbr1	gamma-aminobutyric acid type B receptor subunit 1 isoform X3	1.91	−0.57	40.52	7.59	2.60×10^{-03}	4.32×10^{-01}
	Metabotropic glutamate receptor family	LOC100697448	probable G-protein coupled receptor 158	1.32	−1.60	146.57	18.84	3.60×10^{-04}	2.14×10^{-05}
	Peptide	LOC100712161	delta-type opioid receptor	1.92	−0.68	19.85	3.05	5.22×10^{-03}	4.03×10^{-01}
	Peptide	LOC102077197	galanin receptor type 2	1.19	−3.99	34.33	0.91	5.89×10^{-02}	5.32×10^{-06}
	Peptide	LOC100699280	growth hormone secretagogue receptor type 1 isoform X1	2.26	−1.79	16.17	1.04	5.00×10^{-03}	9.95×10^{-02}
	Peptide	nmbr	neuromedin-B receptor	1.44	−5.06	531.66	5.76	2.17×10^{-02}	1.11×10^{-17}
	Peptide	ntsr1	neurotensin receptor type 1 isoform X3	2.32	−6.88	337.33	0.44	9.41×10^{-20}	3.08×10^{-14}
	Peptide	LOC100702751	prolactin-releasing peptide receptor	2.62	0.80	11.61	3.49	1.61×10^{-02}	4.92×10^{-01}
	Peptide	LOC100692730	prolactin-releasing peptide receptor isoform X1	1.56	−3.28	23.95	1.00	1.90×10^{-02}	7.24×10^{-04}
	Peptide	LOC100534508	putative gonadotropin-releasing hormone II receptor	3.68	−2.69	226.32	2.48	6.94×10^{-17}	1.49×10^{-05}
	Peptide	LOC100696221	relaxin-3 receptor 1-like	1.95	−1.24	11.78	1.63	1.90×10^{-02}	2.32×10^{-01}
	Peptide	sstr3	somatostatin receptor type 3	3.70	2.71	71.43	35.19	8.39×10^{-11}	1.84×10^{-06}
	Peptide	agtr1	type-1 angiotensin II receptor isoform X1	2.17	-5.16	91.38	0.31	1.91×10^{-10}	5.89×10^{-08}
	Secretin receptor family	gcgr	glucagon receptor	1.10	−2.17	58.92	5.79	7.82×10^{-04}	3.24×10^{-08}
	Secretin receptor family	LOC100710530	pituitary adenylate cyclase-activating polypeptide type I receptor isoform X1	1.84	−3.60	324.66	6.14	6.85×10^{-08}	5.46×10^{-19}
	Secretin receptor family	LOC100710985	pituitary adenylate cyclase-activating polypeptide type I receptor isoform X1	1.34	−0.18	69.49	23.48	5.20x10-03	7.46x10-01
	Secretin receptor family	LOC100698743	receptor activity-modifying protein 1 isoform X2	1.42	−1.65	22.35	2.78	3.82×10^{-02}	2.84×10^{-02}
	Vision	LOC100702425	pinopsin isoform X1	1.67	−3.37	73.93	2.48	2.87×10^{-03}	7.87×10^{-07}
	Vision	LOC100695085	vertebrate ancient opsin	1.20	−4.46	122.65	2.32	2.66×10^{-01}	1.55×10^{-06}
FSH	Biogenic amine	htr1b	5-hydroxytryptamine receptor 1B	1.15	1.96	53.99	95.02	1.46×10^{-01}	2.26×10^{-03}
	Adhesion receptor family	adgrb2	adhesion G protein-coupled receptor B2 isoform X5	1.74	2.01	294.70	354.39	3.30×10^{-10}	1.78×10^{-14}
	Adhesion receptor family	LOC100698301	adhesion G protein-coupled receptor L1 isoform X3	1.07	2.87	84.98	294.62	1.99×10^{-03}	7.49×10^{-24}
	Adhesion receptor family	LOC102080577	adhesion G-protein coupled receptor G7 isoform X2	0.08	1.42	6.37	16.19	9.43×10^{-01}	1.65×10^{-02}
	Adhesion receptor family	celsr3	cadherin EGF LAG seven-pass G-type receptor 3 isoform X1	1.06	1.15	665.82	706.36	1.08×10^{-04}	7.56×10^{-06}
	Base and nucleoside	LOC102076535	P2Y purinoceptor 14 isoform X2	−0.77	2.02	2.82	20.13	5.27×10^{-01}	4.25×10^{-03}
	Biogenic amine	drd5	D(1B) dopamine receptor	0.16	2.51	6.23	32.57	8.83×10^{-01}	2.50×10^{-06}
	Frizzled / Smoothened family	fzd5	frizzled-5	−0.17	2.44	6.07	35.77	8.69×10^{-01}	2.23×10^{-07}
	Lipid	LOC100708895	free fatty acid receptor 3-like	−0.41	2.60	3.28	26.82	7.47×10^{-01}	8.24×10^{-05}
	Metabotropic glutamate receptor family	gabbr2	gamma-aminobutyric acid type B receptor subunit 2	0.95	1.66	46.18	76.40	4.52×10^{-02}	1.41×10^{-05}
	Metabotropic glutamate receptor family	gprc5c	G-protein coupled receptor family C group 5 member C isoform X3	0.25	1.07	130.50	229.61	4.86×10^{-01}	1.59×10^{-05}
	Metabotropic glutamate receptor family	LOC100705106	LOW QUALITY PROTEIN: metabotropic glutamate receptor 7	0.12	1.43	11.41	28.61	8.84×10^{-01}	5.32×10^{-04}

	Metabotropic glutamate receptor family	LOC100700221	metabotropic glutamate receptor 4	1.15	1.74	19.02	29.47	1.03x10^{-01}	2.09x10^{-03}
	Metabotropic glutamate receptor family	grm5	metabotropic glutamate receptor 5 isoform X2	1.64	4.30	149.71	938.87	1.13x10^{-03}	4.36x10^{-23}
	Metabotropic glutamate receptor family	gprc5a	retinoic acid-induced protein 3	−0.15	3.33	3.28	36.89	9.25x10^{-01}	6.48x10^{-06}
	Others	gpr135	G-protein coupled receptor 135	0.85	1.25	13.13	17.74	1.53x10^{-01}	6.41x10^{-03}
	Others	gpr135	probable G-protein coupled receptor 153 isoform X1	0.93	1.63	78.19	126.52	5.05x10^{-03}	4.73x10^{-09}
	Others	LOC100702509	probable G-protein coupled receptor 173	0.82	1.38	135.29	199.02	1.03x10^{-02}	2.83x10^{-07}
	Others	gpr85	probable G-protein coupled receptor 85	1.56	2.54	83.96	166.59	4.42x10^{-07}	3.97x10^{-20}
	Peptide	LOC100700667	cholecystokinin receptor	2.62	7.92	511.82	20112.98	1.05x10^{-16}	1.31x10^{-159}
	Peptide	LOC100534509	GnRH receptor type2	0.18	2.26	11.63	48.04	8.48x10^{-01}	2.74x10^{-06}
	Peptide	LOC100693278	melanocortin receptor 5	0.19	1.67	11.93	32.20	8.80x10^{-01}	2.22x10^{-02}
	Peptide	npffr2	neuropeptide FF receptor 2	0.95	2.42	16.49	47.97	1.52x10^{-01}	4.66x10^{-07}
	Peptide	gpr37	prosaposin receptor GPR37	0.25	1.81	28.96	84.17	7.03x10^{-01}	3.88x10^{-06}
	Secretin receptor family	LOC100710271	pituitary adenylate cyclase-activating polypeptide type I receptor	−0.97	1.64	3.32	19.55	2.86x10^{-01}	1.42x10^{-03}
	Vision	LOC100690289	melanopsin-A	0.04	2.06	28.74	114.54	9.63x10^{-01}	9.10x10^{-06}
LH&FSH	Adhesion receptor family	adgrb3	adhesion G protein-coupled receptor B3 isoform X6	2.35	2.95	114.02	81.29	1.06x10^{-03}	8.13x10^{-02}
	Biogenic amine	LOC100704531	D(4) dopamine receptor	3.26	2.86	326.07	374.24	2.56x10^{-09}	8.86x10^{-12}
	Lipid	ptgfr	prostaglandin F2-alpha receptor	2.28	0.58	9.08	13.13	3.12x10^{-02}	1.43x10^{-03}
	Metabotropic glutamate receptor family	LOC100695498	gamma-aminobutyric acid type B receptor subunit 1 isoform X1	1.73	2.06	370.19	464.45	1.78x10^{-17}	1.40x10^{-26}
	Metabotropic glutamate receptor family	LOC100712211	G-protein coupled receptor family C group 5 member C isoform X2	1.88	2.13	41.02	48.16	1.09x10^{-04}	1.69x10^{-06}
	Metabotropic glutamate receptor family	gpr158a	probable G-protein coupled receptor 158 isoform X2	1.63	1.73	241.50	256.28	4.73x10^{-15}	2.49x10^{-18}
	Others	LOC100696877	G-protein coupled receptor 3	1.06	0.57	44.62	13.23	6.40x10^{-05}	3.70x10^{-01}
	Peptide	LOC100695300	neuropeptides B/W receptor type 2	2.14	2.33	125.11	118.99	1.06x10^{-04}	1.04x10^{-04}
	Peptide	XP_005475771.1	probable G-protein coupled receptor 19	1.46	1.82	51.75	68.00	2.99x10^{-06}	9.15x10^{-11}
	Peptide	LOC100691599	relaxin-3 receptor 1	1.55	1.46	25.89	18.78	5.03x10^{-05}	2.10x10^{-04}

Table S5. GPCRs identified in BRITE functional analysis in females.

				log2FoldChange		Av_Norm		Padj	
	BRITE Functional Hierarchies	Gene ID	Protein Name	LH.VS.neg	FSH.VS.neg	LH	FSH	LH.VS.Neg	FSH.VS.Neg
LH	Adhesion receptor family	LOC100705251	adhesion G protein-coupled receptor B1 isoform X2	1.02	−6.16	1123.72	8.17	3.14x10^{-03}	6.46x10^{-55}
	Adhesion receptor family	LOC102077352	adhesion G-protein coupled receptor G1	1.68	−4.73	29.68	0.23	9.37x10^{-03}	1.63x10^{-04}
	Base and nucleoside	LOC100692921	P2Y purinoceptor 12	2.36	−2.95	13.92	0.23	3.07x10^{-02}	5.74x10^{-02}
	Base and nucleoside	p2ry6	P2Y purinoceptor 6 isoform X1	1.98	−1.46	54.21	5.40	4.53X10^{-04}	3.17X10^{-02}

	Lipid	LOC100701533	G-protein coupled receptor 84	1.80	−4.69	18.89	0.00	5.14×10^{-03}	1.88×10^{-04}
	Lipid	LOC102080433	prostaglandin E2 receptor EP1 subtype isoform X2	1.09	−5.59	81.81	0.58	3.40×10^{-03}	1.05×10^{-09}
	Biogenic amine	LOC100534567	muscarinic receptor 4 subtype isoform X1	1.55	−1.02	35.40	6.06	4.53×10^{-04}	6.83×10^{-02}
	Biogenic amine	drd2	D(2) dopamine receptor isoform X1	1.25	0.29	36.17	17.65	1.99×10^{-03}	5.48×10^{-01}
	Chemokine	LOC100708026	C-C chemokine receptor type 3	1.12	−2.69	29.67	1.80	4.47×10^{-02}	1.57×10^{-04}
	Chemokine	cmklr1	chemokine-like receptor 1	3.14	−1.31	12.63	1.20	8.66×10^{-03}	4.48×10^{-01}
	Chemokine	gpr1	G-protein coupled receptor 1	1.74	−6.80	78.11	0.00	2.25×10^{-04}	5.85×10^{-09}
	Chemokine	LOC100711803	chemokine XC receptor 1	1.59	−3.82	33.71	0.00	1.71×10^{-06}	5.60×10^{-03}
	Chemokine	LOC100691705	chemokine XC receptor 1-like isoform X1	3.49	−3.95	71.86	1.28	3.53×10^{-04}	1.61×10^{-07}
	Others	LOC100698978	integral membrane protein GPR137B	1.83	−0.36	17.04	3.70	1.55×10^{-02}	7.10×10^{-01}
	Peptide	agtr1	type-1 angiotensin II receptor isoform X1	1.57	−6.55	58.63	0.00	7.70×10^{-03}	4.70×10^{-08}
	Peptide	LOC100534508	putative gonadotropin-releasing hormone II receptor	3.10	−3.36	70.78	1.07	5.01×10^{-13}	8.01×10^{-04}
	Peptide	LOC102077197	galanin receptor type 2	1.49	−4.08	26.91	0.89	1.16×10^{-02}	3.68×10^{-04}
	Peptide	ntsr1	neurotensin receptor type 1 isoform X3	1.52	−6.30	302.81	1.57	8.52×10^{-04}	6.31×10^{-16}
	Secretin receptor family	gcgr	glucagon receptor	1.20	−2.63	41.49	3.04	4.25×10^{-03}	7.57×10^{-06}
	Secretin receptor family	LOC100710530	pituitary adenylate cyclase-activating polypeptide type I receptor isoform X1	1.41	−6.08	282.78	1.44	1.43×10^{-03}	5.47×10^{-17}
	Secretin receptor family	ramp2	receptor activity-modifying protein 2 isoform X1	1.54	−4.17	40.08	0.53	4.31×10^{-02}	1.37×10^{-04}
	Vision	LOC100702425	pinopsin isoform X1	1.07	−6.36	36.56	0.00	2.91×10^{-01}	5.22×10^{-07}
**FSH**	Biogenic amine	htr1b	5-hydroxytryptamine receptor 1B	0.42	1.70	21.62	53.24	7.23×10^{-01}	9.91×10^{-04}
	Adhesion receptor family	LOC100698301	adhesion G protein-coupled receptor L1 isoform X3	−0.43	1.92	30.80	149.61	3.07×10^{-01}	2.08×10^{-18}
	Adhesion receptor family	LOC102080577	adhesion G-protein coupled receptor G7 isoform X2	−0.44	1.92	3.70	19.07	8.01×10^{-01}	1.01×10^{-03}
	Base and nucleoside	LOC102076535	P2Y purinoceptor 14 isoform X2	−1.86	3.31	0.57	26.76	3.44×10^{-01}	2.19×10^{-05}
	Biogenic amine	drd5	D(1B) dopamine receptor	−1.53	2.08	2.35	26.53	1.60×10^{-01}	1.86×10^{-04}
	Chemokine	LOC100707345	C-X-C chemokine receptor type 2	0.00	1.37	15.69	37.85	9.98×10^{-01}	1.00×10^{-03}
	Lipid	LOC100708895	free fatty acid receptor 3-like	1.06	5.21	0.60	14.05	7.83×10^{-01}	3.74×10^{-05}
	Metabotropic glutamate receptor family	gabbr2	gamma-aminobutyric acid type B receptor subunit 2	0.36	1.06	25.71	41.41	5.75×10^{-01}	9.79×10^{-04}
	Metabotropic glutamate receptor family	LOC100712211	G-protein coupled receptor family C group 5 member C isoform X2	1.34	2.59	9.25	19.96	1.26×10^{-01}	1.63×10^{-05}
	Metabotropic glutamate receptor family	gprc5c	G-protein coupled receptor family C group 5 member C isoform X3	0.10	0.98	62.80	109.78	8.03×10^{-01}	1.83×10^{-05}
	Metabotropic glutamate receptor family	LOC100705106	LOW QUALITY PROTEIN: metabotropic glutamate receptor 7	−2.23	1.68	1.26	19.57	4.31×10^{-02}	2.68×10^{-03}
	Metabotropic glutamate receptor family	LOC100700221	metabotropic glutamate receptor 4	0.93	1.29	12.07	14.88	2.56×10^{-01}	2.39×10^{-02}
	Metabotropic glutamate receptor family	grm5	metabotropic glutamate receptor 5 isoform X2	−0.03	3.83	26.87	392.39	9.85×10^{-01}	2.50×10^{-31}
	Metabotropic glutamate receptor family	gprc5a	retinoic acid-induced protein 3	−1.35	4.03	1.94	67.55	3.83×10^{-01}	4.00×10^{-10}

	Others	gpr12	G-protein coupled receptor 12	0.49	1.01	30.46	42.75	5.67×10^{-01}	3.01×10^{-02}
	Others	gpr153	probable G-protein coupled receptor 153 isoform X1	0.13	1.13	34.82	70.49	8.60×10^{-01}	5.35×10^{-06}
	Others	gpr85	probable G-protein coupled receptor 85	0.59	1.41	41.07	72.74	1.98×10^{-01}	2.93×10^{-06}
	Peptide	LOC100700667	cholecystokinin receptor	1.56	8.85	55.43	8826.10	6.91×10^{-05}	7.88×10^{-174}
	Peptide	LOC100534509	GnRH receptor type2	−1.29	1.99	5.49	52.06	1.65×10^{-01}	4.67×10^{-04}
	Peptide	npffr2	neuropeptide FF receptor 2	0.72	1.05	14.91	19.36	3.59×10^{-01}	3.72×10^{-02}
	Peptide	gpr37	prosaposin receptor GPR37	0.03	2.41	9.96	49.99	9.89×10^{-01}	3.23×10^{-08}
	Peptide	LOC100710302	somatostatin receptor type 5 isoform X3	0.00	2.08	31.65	137.92	9.99×10^{-01}	1.26×10^{-21}
	Secretin receptor family	LOC100710271	pituitary adenylate cyclase-activating polypeptide type I receptor	−0.03	2.11	3.13	13.11	9.93×10^{-01}	3.62×10^{-02}
**LH&FSH**	Biogenic amine	LOC100704531	D(4) dopamine receptor	1.52	1.64	230.68	248.82	6.56×10^{-05}	2.63×10^{-06}
	Adhesion receptor family	adgrb2	adhesion G protein-coupled receptor B2 isoform X5	1.23	0.80	193.34	144.30	4.27×10^{-04}	2.01×10^{-02}
	Metabotropic glutamate receptor family	LOC100695498	gamma-aminobutyric acid type B receptor subunit 1 isoform X1	0.84	1.30	191.00	262.85	9.84×10^{-06}	1.08×10^{-14}

Table S6. Dominant genes of gonadotroph cells that are conserved in rat and tilapia. Values are the average of the normalized read counts in tilapia LH and FSH cells.

	Rat_ID	ZF_ID	Protein Name	Normalized Read Counts			
				Male		Female	
				LH Cells	FSH Cells	LH Cells	FSH Cells
**LH**	Lhb	lhb	gonadotropin subunit beta-2	5653.06	20.29	1334.85	12.76
	KCNMB4	kcnmb2	calcium-activated potassium channel subunit beta-4 isoform X1	276.29	44.56		
	GnRH	gnrhr4	putative gonadotropin-releasing hormone II receptor	226.32	2.48	70.78	1.07
	FOXP2	foxp2	forkhead box protein P2	93.40	54.76		
	CD164L2	cd164l2	CD164 sialomucin-like 2 protein isoform X2	35.01	1.18		
	OTOF	otofb	otoferlin	22.26	3.27		
	TGFBR3L	engl	transforming growth factor beta receptor type 3			14.39	0.83
**FSH**	Fshb	fshb	follitropin subunit beta isoform X1	193.54	48573.70	37.09	30114.06
	KCNH6	kcnh6a	potassium voltage-gated channel subfamily H member 6	519.90	1509.48	305.64	951.50
	TGFBR3	tgfbr3	transforming growth factor beta receptor type 3 isoform X2	46.17	1463.64	21.17	676.10
	OTOF	otofa	otoferlin isoform X6	468.34	1350.29	169.90	938.71
	RFX3	rfx3	transcription factor RFX3	528.46	638.25	172.65	360.11
	KCNA4	kcna4	potassium voltage-gated channel subfamily A member 4	152.66	637.44	80.95	345.96
	CAMTA1	camta1a	calmodulin-binding transcription activator 1	219.79	363.04	107.32	174.30
	AR	Ar-α	androgen receptor alpha			65.14	170.60
	RGS4	rgs4	regulator of G-protein signaling 5	142.28	258.37	41.53	132.67
	VASH2	vash2	vasohibin-2	103.83	231.13	58.92	120.79
	AR	ar	androgen receptor-like	136.69	217.90	29.14	128.77

	CAMTA1	camta1a	calmodulin-binding transcription activator 1 isoform X4	127.42	173.42		
	NTRK3	ntrk3b	NT-3 growth factor receptor isoform X1	46.69	167.79		
	KCNA4	kcna4-p	potassium voltage-gated channel subfamily A member 4, partial	32.62	115.80	14.33	84.77
	AR	ar	androgen receptor	56.07	97.78	16.18	46.90
	AIF1L	aif1l	allograft inflammatory factor 1-like	36.07	80.26	17.59	49.91
	FKBP1B	fkbp1b	peptidyl-prolyl cis-trans isomerase FKBP1B isoform X3	40.48	74.02	17.88	32.12
	GnRHR	gnrhr1	GnRH receptor type2	11.63	48.04	5.49	33.49
	RNF183	rnf183	serine/arginine repetitive matrix protein 1	5.17	15.43	4.16	13.79
**LH & FSH**	GJD2	gjd2b	gap junction delta-2 protein	530.56	675.62	369.08	359.40

Table S7. conserved GPCRs in rat and tilapia gonadotrophs. For the rat values are the mean expression of the single cell gonadotrophs, for the tilapia values are the average of the normalized read counts in LH and FSH cells.

	**Rat ID**	**Rat**		**ZF_ID**	**Tilapia**			
		**Mean Expression**			**Normalized Read Counts**			
		**Male**	**Female**		**LH Cell**	**FSH Cell**	**LH Cell**	**FSH Cell**
**LH**	Gnrhr	8.21	7.57	gnrhr3	226.32	2.48	70.78	1.07
	Adcyap1r1	0.75	0.05	Adcyap1r2	324.66	6.14	282.78	1.44
	Gpr158	0.12	0.06	gpr158	146.57	18.84		
	Sstr3	0.10	0.13	sstr3	71.43	35.19		
	Ramp1		0.12	Ramp1	22.35	2.78		
	Gpr137	0.09	0.12	Gpr137			17.04	3.70
**FSH**	Gprc5c	0.06	0.04	gprc5d(3)	130.50	229.61	62.80	109.78
	Adgrl1	0.26	0.44	adgrl1	84.98	294.62	30.80	149.61
	Gpr85	0.39	0.28	Gpr86	83.96	166.59	41.07	72.74
	Gabbr2	0.11	0.06	Gabbr2	46.18	76.40	25.71	41.41
	Gabbr1	0.16	0.19	gabbr1(3)	40.52	7.59	73.83	173.49
	Gnrhr	8.21	7.57	gnrhr1	11.63	48.04	5.49	52.06
	Fzd5	0.08	0.06	Fzd5	6.07	35.77		
	Sstr3	0.10	0.13	sstr5			31.65	137.92
**LH&FSH**	Celsr3	0.10	0.03	Celsr3	665.82	706.36		
	Gabbr1	0.16	0.19	gabbr1(1)	370.19	464.45	191.00	262.85
	Gpr158	0.12	0.06	gpr158(2)	241.50	256.28		
	Gpr153		0.06	Gpr153	78.19	126.52	34.82	70.49
	Gpr19	0.14	0.19	Gpr19	51.75	68.00		
	Gprc5c	0.06	0.04	gprc5d(2)	41.02	48.16	9.25	19.96

Table S8. Distribution of the number of genes according to the normalized reads (NR) counts in each RNA-seq sample.

Males

Cells	NR > 0 °	0 < NR≤ 1 °	1 < NR ≤ 10 °	10 < NR≤ 100 °	NR > 100 °
**Before**	22,098	494	6,880	9,433	5,291
**LH**	21,880	1,775	5,945	8,667	5,493
**FSH**	21,728	3,582	5,558	7,247	5,341
**Negative**	22,090	356	6,546	9,602	5,586

Females

Vells	NR > 0 °	0 < NR ≤ 1 °	1 < NR ≤ 10 °	10 < NR ≤ 100 °	NR> 100 °
**Before**	22,382	1,716	8,125	9,436	3,105
**LH**	22,179	2,399	7,364	9,032	3,384
**FSH**	20,472	4,281	5,388	7,449	3,354
**Negative**	22,275	1,641	7,877	9,418	3,339
